# Lack of Sex Differences in Psychostimulant-Induced Locomotor Activity When Comparing Rats From the Same Behavioral Groups

**DOI:** 10.1016/j.bpsgos.2025.100519

**Published:** 2025-04-25

**Authors:** Anthony M. Tigano, Martin O. Job

**Affiliations:** Department of Biomedical Sciences, Cooper Medical School of Rowan University, Camden, New Jersey

**Keywords:** Cocaine, Locomotor activity, Median-split analysis, Normal mixtures clustering, Psychostimulant, Sex differences

## Abstract

**Background:**

We hypothesized that sex differences in baseline-, saline-, and psychostimulant-induced locomotor activity (LMA) would not be observed when we compared males and females that belonged to the same behavioral group(s). Our aim was to determine whether we could detect differences between males and females within a truly distinct behavioral group. To identify behavioral groups, current models use median-split analysis typically of one variable, but this procedure may not be effective. With the rationale that clustering analysis of several variables is a more robust grouping strategy, we developed a new model termed the MISSING (Mapping Intrinsic Sex Similarities as an Integral quality of Normalized Groups) model to test our hypothesis.

**Methods:**

We obtained baseline LMA, drug-induced LMA, and a new variable that integrates both baseline and drug-induced LMA (drug-induced LMA normalized-to-baseline activity-time) from LMA assessments following saline (males *n* = 12, females *n* = 11) and cocaine 10 mg/kg (male *n* = 22, female *n* = 23) intraperitoneal injections, and intra–nucleus accumbens dopamine 15 μg/side (male *n* = 20, female *n* = 17). Using regression analysis and analysis of variance, we compared the MISSING model with the current model for effectiveness in identifying distinct groups.

**Results:**

The MISSING model was superior to the median split in distinct behavioral group identification. For both models, we confirmed that there were no differences in psychostimulant-induced LMA when we compared males and females within the same group/cluster.

**Conclusions:**

There were no sex differences in psychostimulant-induced LMA when we compared rats from the same behavioral group(s).

## Plain Language Summary

We hypothesized that there would be no sex differences in baseline and psychostimulant-induced locomotor activity when we compared males and females from the same behavioral groups. Current models to identify distinct groups rely on median-split analysis of these variables, but as this may not represent the most effective procedure, we developed the new MISSING model. The MISSING model includes normal clustering analysis of baseline locomotor activity, drug-induced locomotor activity, and a variable that integrates both baseline and drug-induced locomotor activity (drug-induced locomotor activity normalized-to-baseline) to define similar/different behavioral groups of males and females. We obtained the variables mentioned above from experiments designed to assess saline-, cocaine-, and dopamine-induced locomotor activity. We used the MISSING model to identify distinct behavioral clusters and to determine the effect of these behavioral clusters on sex differences. We also compared the new MISSING model with the current model (median split), and we determined that the MISSING model was more effective than the median split at identifying distinct clusters. We confirmed our hypothesis: there were no differences in the behavioral variables when we compared males and females within the same cluster. Sex differences appeared to be observed when we compared males and females from different behavioral clusters. Differences between males and females in drug-induced effects may not be solely due to biological sex, as is commonly assumed, but rather defined by behavioral group identity.

**Figure 1:**
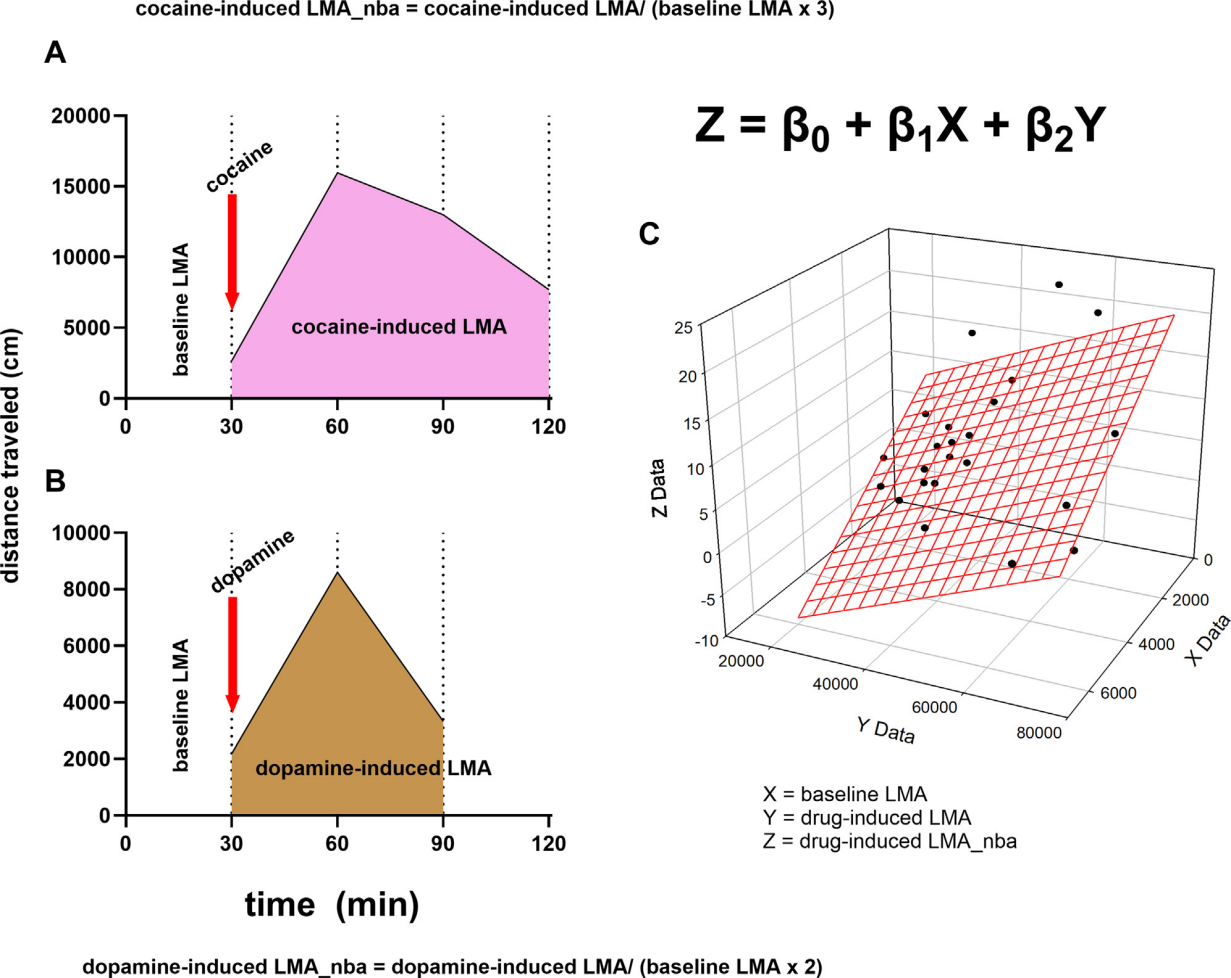
Variables of interest. The figure shows how we derived 1) the baseline activity–time normalized variable for cocaine (or saline)- and dopamine-induced LMA (drug-induced LMA_nba) and 2) the variables defining the interaction between baseline LMA, drug-induced LMA, and drug-induced LMA_nba in a 3D space. For **(A)** (cocaine or saline) and **(B)** (dopamine), the x-axis represents time (minutes), and the y-axis represents the distance traveled (cm). For cocaine and/or saline **(A)**, drug-induced LMA was estimated for 90 minutes after 30 minutes of baseline LMA, and termed cocaine-induced LMA (corresponding to the area under the curve) and cocaine-induced LMA_nba was calculated as cocaine-induced LMA / (baseline LMA × *N*), where *N* = time required for drug-induced LMA / time required for baseline LMA. In the case of cocaine **(A)**, *N* = 3. For dopamine **(B)**, drug-induced LMA was estimated for a duration of 60 minutes after 30 minutes of baseline LMA, and termed dopamine-induced LMA (corresponding to the area under the curve). Dopamine-induced LMA_nba was calculated as dopamine-induced LMA / (baseline LMA × 2). We also derived variables that define the relationships (behavior interaction complex) between these 3 variables on a 3D plane **(C)**. To do this, we conducted multiple linear regressions to derive additional variables (β_0_, β_1_, and β_2_) as shown in the equation below (equation 3): *Z* = β_0_ + β_1_*X* + β_2_*Y*, where variable *Z* = drug-induced LMA_nba, variable *X* = baseline LMA, variable *Y* = drug-induced LMA, β_0_ = *Z*-plane intercept variable, β_1_ = slope of the relationship between *X* and *Z*, β_2_ = slope of the relationship between *Y* and *Z*. 3D, 3-dimensional; LMA, locomotor activity; LMA_nba, LMA normalized-to-baseline activity-time.

Utilizing sex as a biological variable (1, 2, 3, 4, 5, 6) is important for a clear understanding of the mechanisms of sex differences in psychostimulant-related behavioral effects (7, 8, 9, 10). It is generally accepted that there are sex differences in psychostimulant-induced locomotor activity (LMA), with females showing greater responses than males (11, 12, 13, 14, 15, 16, 17, 18, 19, 20, 21, 22, 23, 24, 25, 26, 27, 28, 29, 30, 31, 32, 33, 34, 35, 36, 37, 38, 39, 40), but this is not always the case (13,26,34,41, 42, 43, 44, 45, 46, 47). In addition to sex differences in psychostimulant-induced LMA per se, sex differences have also been observed in baseline LMA, with females showing greater LMA than males (19, 20, 21, 22, 23,25, 26, 27,39,42,46,48,49), but not always (18,19,32,37). Because baseline LMA can regulate/predict subsequent drug-induced effects, including psychostimulant-induced LMA (19,48,50, 51, 52, 53, 54), we derived a normalized variable that accounts for the link between baseline LMA and drug-induced LMA. We termed this new variable drug-induced LMA normalized-to-baseline activity-time (LMA_nba) (Figure 1). No studies have examined this normalized variable for sex differences.

Because males and females do not represent behaviorally homogeneous populations (45,55, 56, 57), the inconsistencies in the observation of sex differences in psychostimulant-induced behavioral effects may be due to comparisons of males and females from distinct behavioral groups. When distinct behavioral types/groups are compared, sex differences are constrained by the behavioral group of males and females being compared (Table S1). To identify distinct behavioral groups, current methods in the field use the median-split procedure to separate rats into high-response versus low-response groups, corresponding to behavioral responses above and below the median of the population, respectively (58, 59, 60). However, because the median split focuses on data above and below the median, it is likely to be ineffective at 1) distinguishing rats with responses close to the median and 2) identifying more than two behavioral groups. To address these limitations, normal mixture clustering may be used (61). Normal mixture clustering is an unbiased approach for the identification of subgroups based on how similar, or different, they are with regard to several variables.

Based on the evidence presented in Table S1, we hypothesized that there would be no differences in baseline LMA, saline-induced LMA, and drug-induced LMA when males and females from the same behavioral group/cluster were compared. Because of the limitations of the median-split procedure in identifying distinct behavioral groups, we developed a model termed the MISSING (Mapping Intrinsic Sex Similarities as an Integral quality of Normalized Groups) model (Figure S1). The MISSING model integrates the 3 variables (baseline LMA, drug-induced LMA, and the new drug-induced LMA_nba) (Figure 1) using normal mixture clustering analysis to identify distinct behavioral clusters. The MISSING model proposes that for psychostimulant-induced LMA, 1) there will be no sex differences when we compare males and females within the same behavioral cluster; 2) sex differences will be observed mostly when we compare males and females from different behavioral clusters; and 3) even if we detect sex differences between males and females in the same behavioral cluster, these differences will not be as significant as differences between males and females from different clusters. In other words, the MISSING model suggests that behavioral differences between males and females are not exclusively driven by biological sex.

The MISSING model has not been tested. Using the MISSING model in rats, we tested our hypothesis for LMA following intraperitoneal (i.p.) administration of saline (vehicle) and cocaine (a psychostimulant) and intra–nucleus accumbens (NAc) injections of dopamine (thought to be the proposed mechanism for how psychostimulants increase LMA). We also compared the MISSING model with the current model (median split) to determine which was more effective at identifying distinct behavioral groups.

## Methods and Materials

Experiments and animal care were carried out in accordance with the Institute of Animal Care and Use Committee of Emory University and followed the guidelines outlined in the National Institutes of Health Guide for the Care and Use of Laboratory Animals. We used a total of 105 age-matched male and female adult Sprague Dawley rats (Charles River Laboratories). Rats were acclimatized to the housing facility for at least 1 week before any experimental procedures were conducted. They were given rat chow and water ad libitum and maintained on a 12-hour light/dark cycle (lights on at 7 am). We conducted all experiments between 10 am and 6 pm. The saline and cocaine experiments were conducted at the same time but in different rats, while the dopamine experiments were conducted at a different time.

### Surgery

For the dopamine experiments, the rats underwent surgery. Surgeries were done as previously described (14,62,63). Briefly, under isoflurane inhalation anesthesia or with a cocktail of ketamine HCl (Fort Dodge Animal Health) and dexmedetomidine HCl (Orion Corporation) injection anesthesia, stereotaxic surgical procedures were utilized to implant rats with bilateral stainless steel guide cannulae (22 gauge; P1 Technologies) placed to access the NAc core. The target coordinates (from bregma) for accessing the NAc core were as follows: anteroposterior (AP) = 1.6 mm, mediolateral (ML) = ±1.5 mm, and dorsoventral (DV) = −5.7 mm (64). The guide cannulae assembly was secured to the skull using 2 to 4 stainless steel screws, glue, and dental cement. To prevent cannulae blockage, a bilateral obturator extending 0.5 mm past the tip of the cannulae was inserted into the guide cannulae. All rats were allowed to recover for at least 1 week before locomotor activity assessments.

### Habituation

Following recovery from surgery, the animals were prepared for infusions and LMA measurement. One to 2 days before testing, animals were placed in the photocell cages so that they could explore the testing chambers to habituate them to experimental conditions. This habituation procedure was done for approximately 30 minutes after rats were briefly handled.

### Assessments of LMA

LMA was measured as distance traveled in centimeters. These assessments were done as in previous studies (14,62,63,65, 66, 67, 68) using locomotor chambers that included transparent Plexiglas walls (dimensions of 40 × 40 × 30 cm) and a photocell cage containing 32 photobeams located 5 cm above the floor (Omnitech Electronics). LMA was analyzed with Digipro software (Omnitech Electronics). On the day of the experiment, rats were placed into the locomotor chambers for 30 minutes to habituate them again (see Habituation details above) to their surroundings before the experiment began. For the experiment, baseline LMA was assessed for 30 minutes. After this, rats were removed from the chambers and administered the appropriate treatment. These treatments were 1) saline, 2) cocaine (10 mg/kg) (both via the i.p. route), and 3) dopamine (15 μg /0.5 μL bilaterally) via direct injections into the NAc core using a microinjector equipment. After treatments, rats were placed back into the chambers for additional recording of LMA for 90 minutes (for saline and cocaine experiments) and 60 minutes (for dopamine experiments).

### Perfusions and Histology

For rats that received intra-NAc injections, perfusions were done as previously described (14,62,63). After Nissl staining, a stereotaxic brain atlas (64) was used to approximate the bilateral placement of the injector tips for every individual.

### Variables

For these experiments, LMA was obtained for 30 minutes (baseline LMA), after which drug (saline and cocaine [10 mg/kg i.p.] and dopamine [15 μg/side]) were injected, and LMA was assessed for an additional 90 minutes (for saline and cocaine) and 60 minutes (for dopamine) (see Figure 1). To allow comparisons between drug-induced LMA_nba, we adjusted for time by using the following equations:

(1) $$\text{Cocaine}\;-\;\text{induced}\;\text{LM}\mathrm{A\_}\text{nba}\;=\;\frac{\text{cocaine}\;-\;\text{induced}\;\text{LMA}\;\left(90\;\min\right)}{\text{baseline}\;\text{LMA}\;\left(30\;\min\right)\;\times \;3}$$

(2) $$\text{Dopamine}\;-\;\text{induced}\;\text{LMA}\text{\_nba}\;=\;\frac{\text{dopamine}\;-\;\text{induced}\;\text{LMA}\;\left(60\;\min\right)}{\text{baseline}\;\text{LMA}\;\left(30\;\min\right)\;\times \;2}$$

### Additional Variables

Utilizing multiple linear regression analysis, we explored the relationship(s) between drug-induced LMA_nba (as the dependent variable) and baseline LMA and drug-induced LMA (as independent variables) on a 3-dimensional (3D) plane (Figure 1). This analysis yielded additional variables (β_0_, β_1_, and β_2_) as shown in the equation below:

(3) $$Z=\beta_{0}+\beta_{1}X+\beta_{2}Y$$

where variable *Z* = drug-induced LMA_nba, variable *X* = baseline LMA, variable *Y* = drug-induced LMA, β_0_ = *Z*-plane intercept variable, β_1_ = the slope of the relationship between *X* and *Z*, and β_2_ = the slope of the relationship between *Y* and *Z*.

### Power Analysis

Because the normalized variable is derived from both baseline LMA and cocaine-induced LMA (see equations 1 and 2), we set this as a dependent variable and conducted multiple linear regression analysis, which yielded a correlation coefficient = 0.6909. With H_1_ ρ^2^ = 0.6909, H_0_ ρ^2^ = 0, we determined that a sample size (per sex) of 12 rats would be sufficient to power the study with 80% power to detect a difference of 20% in the relationships between variables with a type I error rate of 5%. A comprehensive analysis of clustering models suggests that *n* = 20 is sufficient for power analysis (61), provided there is no overlap between the clusters being identified.

### Statistical Analysis

GraphPad Prism version 10 (GraphPad Software), SigmaPlot version 14.5 (Systat Software Inc.), and JMP Pro version 18 (SAS Institute Inc.) were used for statistical analysis. G∗Power version 3.1.9.7 (69,70) was used for power analysis. Data were expressed as mean ± SEM. We compared the baseline LMA, drug-induced LMA, and drug-induced LMA_nba of males and females using unpaired *t* tests. We utilized normal mixture clustering of these 3 variables for all rats irrespective of sex to identify distinct behavioral groups (with distinctions confirmed using multiple and simple regression analysis, unpaired *t* tests, and/or 1-way analysis of variance [ANOVA]). Thereafter, we used 2-way ANOVA to determine whether there were main effects of sex, cluster, and a sex × cluster interaction. We compared the groups identified using the MISSING model with the groups identified using median-split analysis (current model). Statistical significance was set at *p* < .05 for all analyses, with Tukey’s post hoc test used when significance was detected.

## Results

### Assessments of Sex Differences in Baseline LMA, Drug-Induced LMA, and Drug-Induced LMA_nba

**Figure 2:**
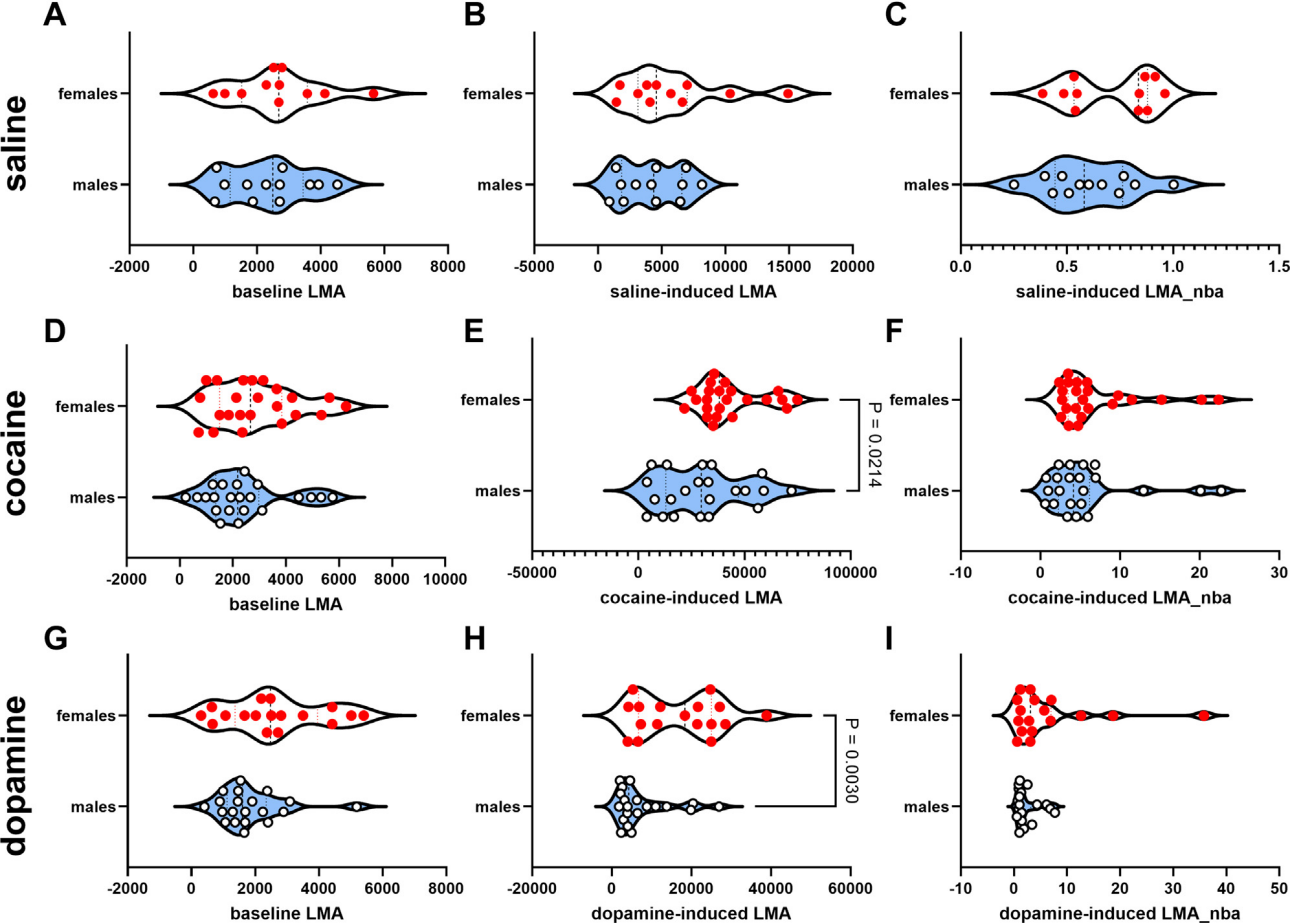
Sex differences/similarities for baseline LMA, drug-induced LMA, and drug-induced LMA_nba. Panels **(A–C)** represent data for males vs. females for comparisons, using unpaired *t* tests, of the variables after saline treatments. Panels **(D–F)** and **(G–I)** are for the same comparisons after cocaine and dopamine treatment, respectively. There were sex differences for cocaine- and dopamine-induced LMA (*p* < .05) but not for any other variables. LMA, locomotor activity; LMA_nba, LMA normalized-to-baseline activity-time.

The histological placements of the NAc injection sites are shown in Figure S2. The values obtained for baseline LMA, drug-induced LMA, and drug-induced LMA_nba are shown in Table 1 separately for males and females. For saline experiments, unpaired *t* tests revealed no significant difference between sexes on baseline LMA (*p* = .5997) (Figure 2A), saline-induced LMA (*p* = .2661) (Figure 2B), and saline-induced LMA_nba (*p* = .2388) (Figure 2C). For cocaine experiments, unpaired *t* tests revealed a significant difference between sexes for cocaine-induced LMA (*p* = .0214) (Figure 2E) but not for baseline LMA (*p* = .3314) (Figure 2D) and/or cocaine-induced LMA_nba (*p* = .4871) (Figure 2F). For dopamine experiments, unpaired *t* tests revealed no significant difference between sexes on baseline LMA (*p* = .0793) (Figure 2G) and dopamine-induced LMA_nba (*p* = .0562) (Figure 2I), but a significant sex difference was observed for dopamine-induced LMA (*p* = .0030) (Figure 2H).

**Table 1:** Baseline LMA, Drug-Induced LMA, and Drug-Induced LMA_nba for Males and Females by Drug (Saline, Cocaine, or Dopamine)

Drug	Baseline LMA, cm	Drug-Induced LMA, cm	Drug-Induced LMA_nba
Saline			
Females, *n* = 11	2681 ± 431	5759 ± 1195	0.7080 ± 0.0629
Males, *n* = 12	2382 ± 363	4208 ± 703	0.6024 ± 0.0603
Cocaine			
Females, *n* = 23	2874 ± 325	42,756 ± 3142	6.9065 ± 1.1524
Males, *n* = 22	2427 ± 319	30,225 ± 4244	5.7272 ± 1.2282
Dopamine			
Females, *n* = 17	2601 ± 373	16,995 ± 2556	6.4184 ± 2.1685
Males, *n* = 20	1830 ± 233	7632 ± 1611	2.3428 ± 0.4896

Values are expressed as mean ± SEM.

LMA, locomotor activity; LMA_nba, LMA normalized-to-baseline activity-time.

### Integrating Variables and Visualizing in 3D

**Figure 3:**
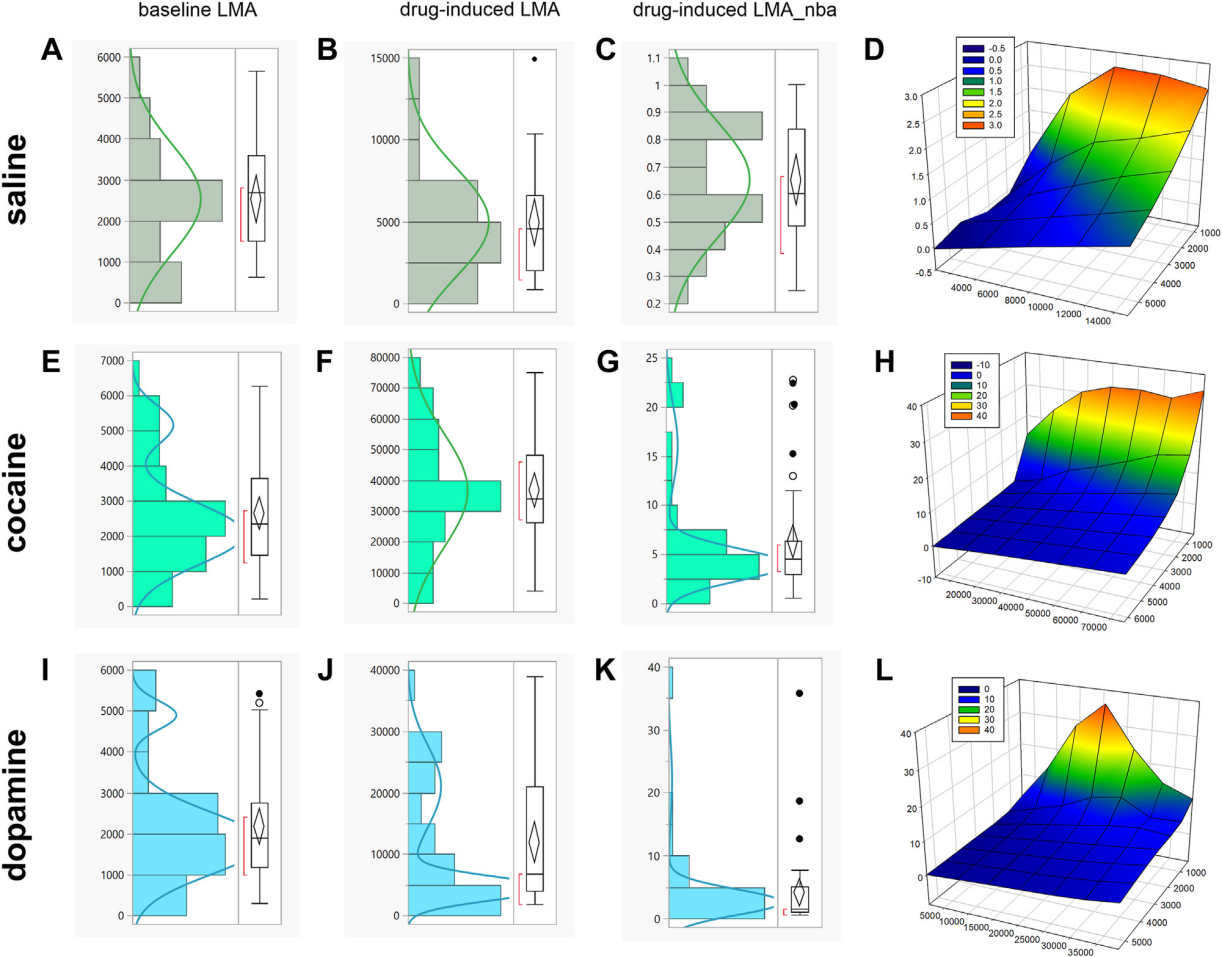
Distribution analysis and 3D visualization of data reveals that there may be more than 1 population of rats in our sample. The graphs **(A–C)** (saline), **(E–G**) (cocaine), and **(I–K)** (dopamine) here represent the distribution analyses of the graphs shown in Figure 2A–C, D–F, and G–I. We integrated all data to visualize a 3D mesh plot, and this procedure revealed population heterogeneity for all drug treatment groups **(D, H, L)**. The implication is that when we simultaneously integrate several variables, there may be more than 1 normally distributed population of rats (males and females) in every drug treatment group. Interestingly, when we detected more than one population of subjects, the median of the population was ineffective in distinguishing them **(E, G,I–K)**. This is the rationale for clustering several variables to identify these normally distributed mixtures. 3D, 3-dimensional; LMA, locomotor activity; LMA_nba, LMA normalized-to-baseline activity-time.

We conducted distribution analysis for all rats (male and female), for all variables, and for all treatments. We observed a normal distribution for saline data (Figure 3A–C) and cocaine-induced LMA (Figure 3F). We observed mixtures of more than 1 normally distributed population for baseline LMA and cocaine-induced LMA_nba (Figure 3E, G). We observed mixtures of more than 1 normally distributed population for all variables for the dopamine group (Figure 3I–K). Interestingly, when we integrated all the data via a 3D plot of baseline LMA, drug-induced LMA, and drug-induced LMA_nba on the x-, y-, and z-axes, we observed that none of the data were homogeneous (see Figure 3D, H, L). Because the data suggest mixtures of more than 1 population of rats, we proceeded with the median-split analysis.

### Sex Differences in Cocaine- and Dopamine-Induced LMA Are No Longer Evident When We Account for Different Behavioral Groups of Males and Females Via Median-Split Analysis

**Figure 4:**
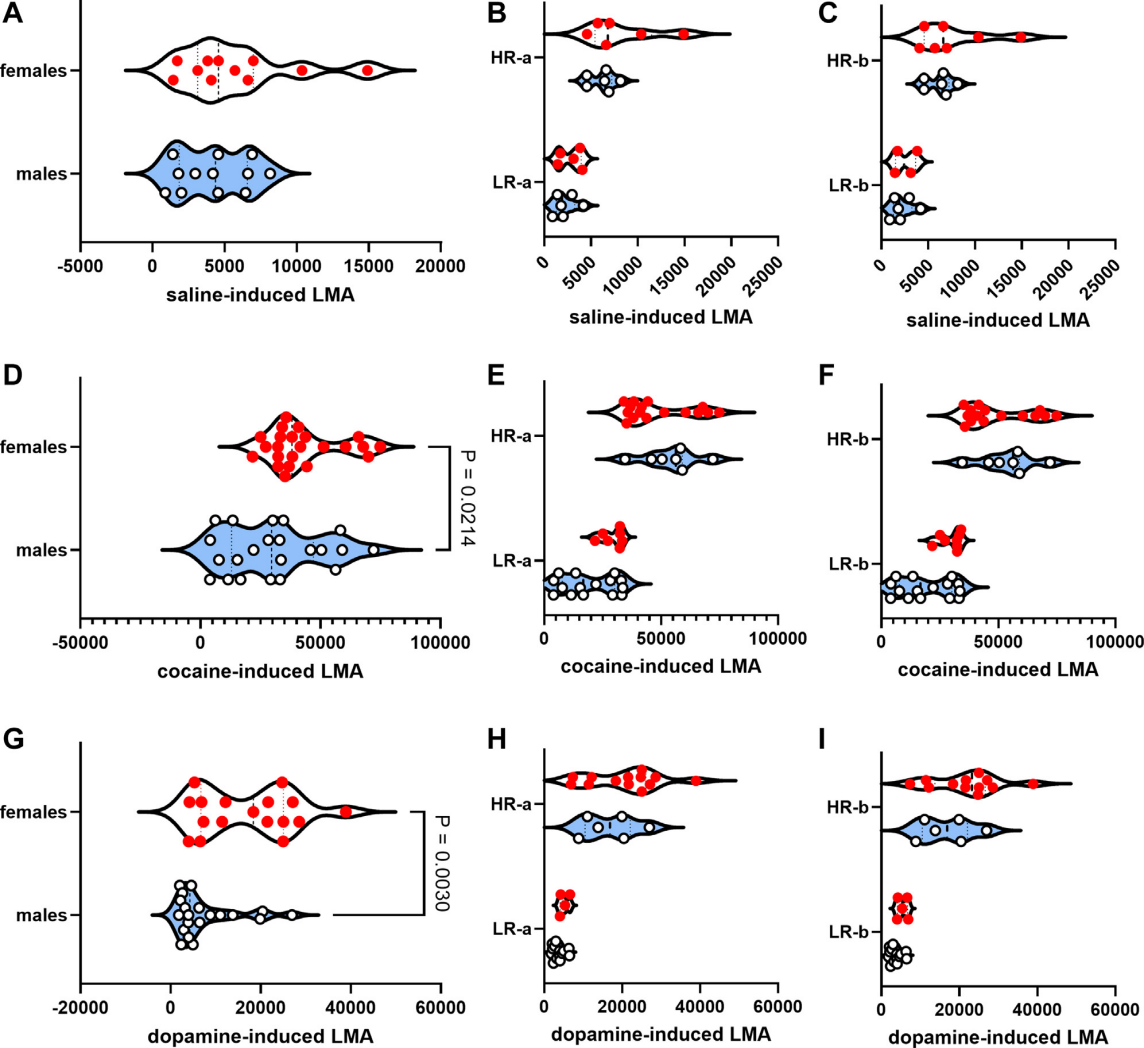
Sex differences in cocaine- and dopamine-induced LMA are no longer evident when we account for different behavioral groups of males and females via median-split analysis. We determined that there were sex differences for cocaine-induced LMA and dopamine-induced LMA but not for the other variables (see Figure 2). These comparisons are reproduced here for saline, cocaine, and dopamine in **(A)**, **(D)**, and **(G)**, respectively. The data in **(A)** are the same as the data in **(B)** and **(C)**. The data in **(D)** is the same as the data in **(E)** and **(F)**. The data in **(G)** are the same as the data in **(H)** and **(I)**. The data in **(A)** are grouped using biological sex (males, females), while **(B)** and **(C)** are grouped using both biological sex and behavioral group (median split-derived groups, LRs vs. HRs). We conducted a median split for all rats and identified LRs and HRs in 2 ways, one in which the median value is the upper limit of the LR group (LR-a and HR-a) and another in which the median is the lower limit of the HR group (LR-b and HR-b). While we had detected sex differences in cocaine- and dopamine-induced LMA **(E, H)**, but not saline-induced LMA **(A)**, there were no sex × group interactions for LR-a vs. HR-a or LR-b vs. HR-b for any drug treatment **(B, C, E, F, H, I)**. For all these comparisons, there were main effects of group (*p* < .05) but no main effect of sex (*p* > .05). In summary, the differences that were observed when males and females were compared were not due to biological sex but rather to behavioral group identity. HR, high responder; LMA, locomotor activity; LMA_nba, LMA normalized-to-baseline activity-time; LR, low responder.

As per our hypothesis, we conducted a median split of all data to separate rats into low responders (LRs) and high responders (HRs). Because groups were identified based on whether the rats scored above or below the median, we conducted 2 types of HR versus LR comparisons, one in which the median value was the upper limit of the LR group (LR-a and HR-a) and another in which the median was the lower limit of the HR group (LR-b and HR-b). We used 2-way ANOVA to determine whether there was a sex (males, females) × group interaction. While we detected sex differences in cocaine- and dopamine-induced LMA (Figure 4D, G), but not saline-induced LMA (Figure 4A), there were no sex × group interactions (*p* > .05) for LR-a versus HR-a or for LR-b versus HR-b for any drug treatment (Figure 4B, C, E, F, H, I). For all these comparisons, there were main effects of group (*p* < .05) but no main effect of sex (*p* > .05).

It should be noted that the choice of applying the median as the upper limit of the LR or the lower limit of the HR can lead to distinct groups of LRs and HRs (see Figures S3 and S4), confirming that, in addition to the limitations already discussed, there is some subjectivity to the median-split procedure. Therefore, we cannot be sure that median split–identified groups are distinct, particularly when several variables are assessed. To address this limitation, we developed/utilized the MISSING model.

### Distinct Behavioral Clusters Identified Via the MISSING Model

**Figure 5:**
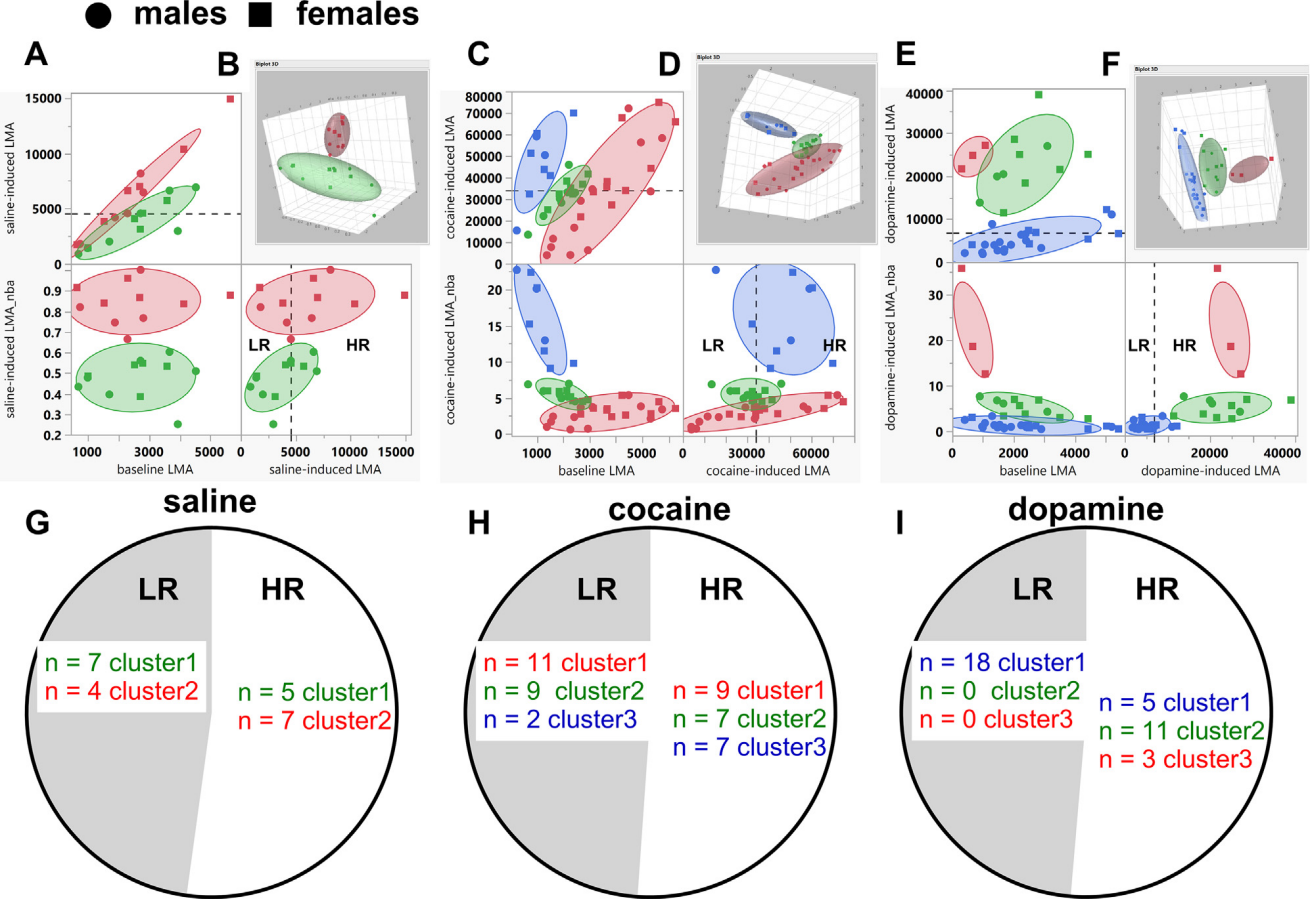
MISSING model (normal mixtures clustering of several variables for all male and female rats). For experiments related to saline (*n* = 23) **(A, B)**, cocaine (*n* = 45) **(C, D)**, and dopamine (*n* = 37) **(E, F)** administration, we conducted normal mixtures clustering of all these 3 variables (baseline LMA, drug-induced LMA, and drug-induced LMA_nba) for all rats. Panels **(B**, **D**, **F)** are visualizations of these clusters in a 3D plane. This analysis yielded 2, 3, and 3 clusters for saline, cocaine, and dopamine, respectively. The revealed clusters consisted of males and females (except cluster 3 for the dopamine experiment, which had only females). Note that the median split cannot correctly identify these clusters; the 2 groups that were identified by median split included mixtures of members of the different groups that were identified via normal mixtures clustering analysis **(G–I)**. The broken lines in the panels **(A, C, E)** are the median split with LR and HR groups above and below this value. Closed circles represent males while closed squares represent females. The clusters are represented by different colors. 3D, 3-dimensional; HR, high responder; LMA, locomotor activity; LMA_nba, LMA normalized-to-baseline activity-time; LR, low responder; MISSING, Mapping Intrinsic Sex Similarities as an Integral quality of Normalized Groups.

Normal mixtures clustering of baseline LMA, saline-induced LMA, and saline-induced LMA_nba of all rats (*n* = 23) revealed 2 clusters, each consisting of males and females (Figure 5A, B). We labeled these as cluster 1 (*n* = 12: males *n* = 7, females *n* = 5) and cluster 2 (*n* = 11: males *n* = 5, females *n* = 6). The broken lines in Figure 5A represent the median of saline-induced LMA. Note that the median-split procedure distinguished 2 groups, as expected, but the groups that were identified via median split of saline-induced LMA represented combinations of individuals from different clusters (Figure 5G).

Normal mixtures clustering of baseline LMA, cocaine-induced LMA, and cocaine-induced LMA_nba revealed 3 clusters, with all clusters consisting of males and females (Figure 5C, D). We labeled these as cluster 1 (*n* = 22: males *n* = 12, females *n* = 10), cluster 2 (*n* = 14: males *n* = 7, females *n* = 7), and cluster 3 (*n* = 9: males *n* = 3, females *n* = 6). The broken lines in Figure 5C represent the median split of cocaine-induced LMA. As with saline, the median split distinguished 2 groups, but these groups included combinations of rats from distinct clusters (Figure 5H).

Normal mixtures clustering of baseline LMA, dopamine-induced LMA, and dopamine-induced LMA_nba revealed 3 clusters, with 2 clusters consisting of males and females and 1 cluster consisting of only females (Figure 5E, F). We labeled these as cluster 1 (*n* = 23: males *n* = 16, females *n* = 7), cluster 2 (*n* = 11: males *n* = 4, females *n* = 7), and cluster 3 (*n* = 3: males *n* = 0, females *n* = 3). The median split of dopamine-induced LMA was not effective at identifying these clusters (Figure 5I). The values that were obtained for the clusters for baseline LMA, drug-induced LMA, and drug-induced LMA_nba are shown in Table 2.

**Table 2:** Baseline LMA, Drug-Induced LMA, and Drug-Induced LMA_nba for Behavioral Clusters by Drug (Saline, Cocaine, or Dopamine)

Drug	Female/Male	Baseline LMA, cm	Drug-Induced LMA, cm	Drug-Induced LMA_nba
Saline				*p* < .0001
Cluster 1, *n* = 12	5/7	2563 ± 363	3689 ± 595	0.4764 ± 0.0281
Cluster 2, *n* = 11	6/5	2483 ± 436	6325 ± 1166	0.8454 ± 0.0292
Cocaine				*p* < .0001
Cluster 1, *n* = 22	10/12	3691 ± 310	35,319 ± 4823	2.9368 ± 0.2749
Cluster 2, *n* = 14	7/7	2013 ± 161	31,985 ± 2187	5.4546 ± 0.2188
Cluster 3, *n* = 9	6/3	1126 ± 202	47,058 ± 5458	15.9860 ± 1.8168
Dopamine				*p* < .0001
Cluster 1, *n* = 23	7/16	2291 ± 301	5132 ± 577	1.3438 ± 0.1570
Cluster 2, *n* = 11	7/4	2371 ± 307	22,719 ± 2266	5.2692 ± 0.5421
Cluster 3, *n* = 3	3/0	680 ± 222	24,538 ± 1582	22.3671 ± 6.9235

Values are expressed as *n* or mean ± SEM. *p* Values for comparison between clusters for the normalized variable are also included.

LMA, locomotor activity; LMA_nba, LMA normalized-to-baseline activity-time.

### The MISSING Model Is More Effective Than the Median Split at Identifying Distinct Behavioral Groups Consisting of Both Males and Females

The groups identified via median split contain members from several clusters identified by the MISSING model (Figure 5). For the clusters shown in Figure 5, we compared the following variables: baseline LMA, drug-induced LMA, drug-induced LMA_nba, β_0_, β_1_, and β_2_ (see variables in Figure 1).

**Figure 6:**
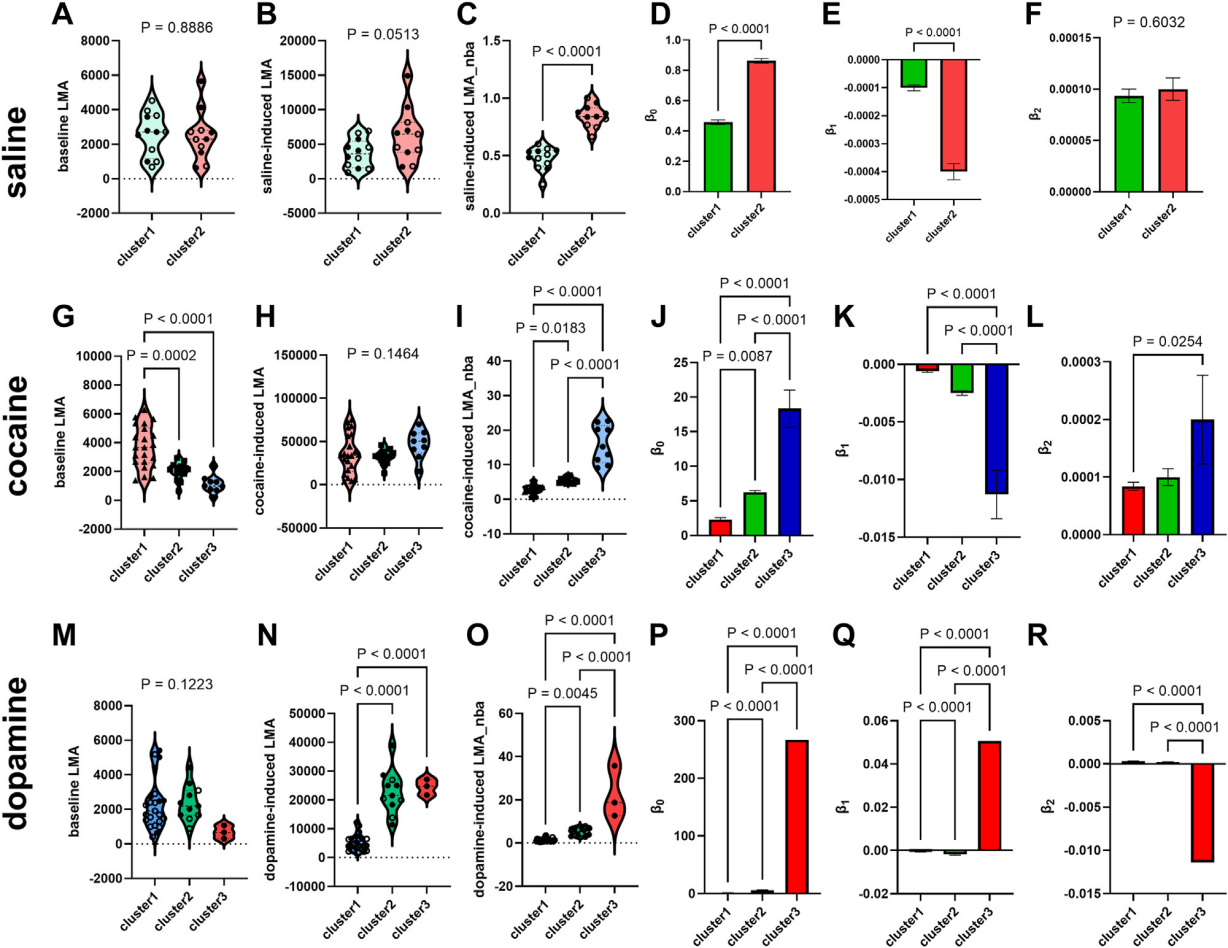
The clusters identified by the MISSING model represent distinct behavioral groups. We compared 6 variables (baseline LMA, drug-induced LMA, drug-induced LMA_nba, β_0_, β_1_, and β_2_) (see variables in Figure 1) between all the clusters identified for each drug treatment (see Figure 5): saline **(A–F)**, cocaine **(G–L)** and dopamine **(M–R)**. For saline, we identified differences (using unpaired *t* tests) between cluster 1 and cluster 2 for saline-induced LMA_nba, β_0_, and β_1_ but not baseline LMA, saline-induced LMA (this approached significant difference: *p* = .0513), and β_2_. Thus, cluster 1 and cluster 2 (saline) were different for 3 of 6 variables (almost 4/6), for a difference for 50% of the variables. For cocaine, we identified differences (using 1-way analysis of variance) between cluster 1, 2, and 3 for all variables except cocaine-induced LMA (*p* > .05). Thus, for clusters 1 to 3 (cocaine), at least 2 of the groups were different for 5/6 variables (83.3%). As with cocaine, clusters 1 to 3 for dopamine-injected rats were different for 83.3% of the variables assessed. Open circles represent males while closed circles represent females. The clusters are represented by different colors. *p* Values are provided in the graphs. After significant differences were detected, comparisons were conducted using Tukey’s post hoc tests. Note that these groups were more different from each other than the groups identified using median split (compare this to the median-split procedure) (Figures S3 and S4). LMA, locomotor activity; LMA_nba, LMA normalized-to-baseline activity-time; MISSING, Mapping Intrinsic Sex Similarities as an Integral quality of Normalized Groups.

For saline, unpaired *t* tests revealed no significant differences between cluster 1 and cluster 2 for baseline LMA (*p* = .8886) (Figure 6A) and saline-induced LMA (*p* = .0513) (Figure 6B) but did reveal significant differences for saline-induced LMA_nba (*p* < .0001) (Figure 6C). Unpaired *t* tests revealed significant differences between cluster 1 and cluster 2 for β_0_ (*p* < .0001) (Figure 6D) and β_1_ (*p* < .0001) (Figure 6E) but not for β_2_ (*p* = .6032) (Figure 6F). For more detailed comparisons, see Figure S5. For saline, cluster 1 and cluster 2 were distinct for 3/6 (50%) variables (see Figure 6A–F and Figure S5). For saline, LR-a versus HR-a were distinct for 4/6 (66.7%) variables while LR-b versus HR-b were also different for 66.7% of the variables analyzed (see Figures S3 and S4).

For cocaine, 1-way ANOVA revealed significant differences between clusters for baseline LMA (*F*_2,24_ = 20.34, *p* < .0001) (Figure 6G) and cocaine-induced LMA_nba (*F*_2,24_ = 82.36, *p* < .0001) (Figure 6I) but not for cocaine-induced LMA (*F*_2,24_ = 2.012, *p* = .1464) (Figure 6H). One-way ANOVA revealed significant differences between clusters 1 to 3 for all 3D plane variables: β_0_ (*F*_2,42_ = 61.09, *p* < .0001) (Figure 6J), β_1_ (*F*_2,42_ = 47.51, *p* < .0001) (Figure 6K), and β_2_ (*F*_2,42_ = 3.818, *p* = .0300) (Figure 6L). For more detailed comparisons, see Figure S6. For cocaine, at least 2 of the 3 clusters identified via the MISSING model were distinct for 5/6 (83.3%) variables (see Figure 6G–L and Figure S6). For cocaine, LR-a versus HR-a and LR-b versus HR-b were distinct for 2/6 (33.3%) variables (see Figures S3 and S4).

For dopamine, 1-way ANOVA revealed no significant differences between clusters for baseline LMA (*F*_2,34_ = 2.237, *p* = .1223) (Figure 6M) but did reveal significant differences between clusters for dopamine-induced LMA (*F*_2,34_ = 64.04, *p* < .0001) (Figure 6N) and dopamine-induced LMA_nba (*F*_2,34_ = 60.87, *p* < .0001) (Figure 6O). One-way ANOVA revealed significant differences between clusters 1 to 3 for all 3D plane variables: β_0_ (*F*_2,34_ = 995.8, *p* < .0001) (Figure 6P), β_1_ (*F*_2,34_ = 294.4, *p* < .0001) (Figure 6Q), and β_2_ (*F*_2,34_ = 640.0, *p* < .0001) (Figure 6R). The differences between clusters could not be explained by dopamine injection site coordinates. One-way ANOVA revealed the following results: AP (*F*_2,34_ = 0.5621, *p* = .5752), ML (*F*_2,34_ = 0.8245, *p* = .4470), and DV (*F*_2,34_ = 0.1176, *p* = .8894). For more detailed comparisons, see Figure S7. For dopamine, at least 2 of the 3 clusters identified via the MISSING model were distinct for 5/6 (83.3%) variables (see Figure 6M–R and Figure S7). For dopamine, LR-a versus HR-a and LR-b versus HR-b were distinct for 3/6 (50%) variables (see Figures S3 and S4).

In summary, the MISSING model identified more distinct behavioral groups (2+) than the median-split model (limited to 2) (see above).

### Sex Differences in Cocaine- and Dopamine-Induced LMA Are No Longer Evident When We Account for Different Behavioral Groups of Males and Females Via the MISSING Model

We wanted to know whether there were sex differences in baseline LMA, cocaine-induced LMA, and cocaine-induced LMA_nba when we accounted for the clusters. For this, we utilized a 2-way ANOVA with sex (males and females) and cluster (cluster 1, cluster 2, and cluster 3) as factors. We did not detect any sex × cluster interactions. The statistics for this analysis are shown in Table S2.

For the dopamine study, we excluded cluster 3 from this specific analysis because it consisted of only females (see Figure 5E, F). For baseline-induced LMA, 2-way ANOVA did not reveal a sex × cluster interaction (*F*_1,30_ = 0.3294, *p* = .5703) or a main effect of cluster (*F*_1,30_ = 0.5182, *p* = .4772) but did reveal a main effect of sex (*F*_1,30_ = 6.676, *p* = .0149). For dopamine-induced LMA, 2-way ANOVA did not reveal a sex × cluster interaction (*F*_1,30_ = 2.051, *p* = .6539) or a main effect of sex (*F*_1,30_ = 2.690, *p* = .1114) but did reveal a main effect of cluster (*F*_1,30_ = 82.50, *p* < .0001). Similarly, for dopamine-induced LMA_nba, 2-way ANOVA did not reveal a sex × cluster interaction (*F*_1,30_ = 2.973, *p* = .0949) or a main effect of sex (*F*_1,30_ = 3.450, *p* = .0731) but did reveal a main effect of cluster (*F*_1,30_ = 89.72, *p* < .0001).

## Discussion

Based on several reports from the literature, we hypothesized that there would be no sex differences when we compared males and females from the same behavioral groups. But to test this hypothesis, we had to develop a model that was effective at identifying distinct behavioral groups relative to the limitations of the currently used median-split procedure. From baseline-dependency principles, we derived a new LMA variable normalized to baseline LMA (and time) to account for a relationship between baseline LMA and drug-induced LMA. Thus, we developed the MISSING model, which utilizes unbiased normal mixtures clustering of several variables, including the new variable, to identify distinct groups. To test our hypothesis, we conducted experiments to assess LMA in male and female rats following injections of saline and cocaine via the i.p. route and injections of dopamine directly into the NAc.

We determined that there were sex differences in cocaine- and dopamine-induced LMA but not saline-induced LMA, consistent with several accounts in the literature. Interestingly, the observed sex differences in psychostimulant-induced LMA were not observed when we examined the new normalized variable, suggesting that further analysis should be done to ascertain that the sex differences detected were related to biological sex. After careful analysis of data distribution and 3D visualization of the simultaneous integration of the 3 variables, we suspected that the sex differences observed for cocaine- and dopamine-induced LMA might be confounded by heterogeneity in the population of rats: There appear to be different populations of males and females in our sample.

We accounted for these behavioral groups in 2 ways, namely 1) via median split and 2) using our new MISSING model (clustering analysis). Using median split, we identified LRs and HRs and determined that there were no sex differences when we compared males and females that belonged to the same behavioral group. We compared the MISSING model with the current approaches that use median split of drug-induced LMA, and we determined that the MISSING model was superior to the current model in its ability to identify more distinct groups. Using the MISSING model, we identified distinct behavioral groups and determined that there were no differences in behavior when we compared males and females from the same behavioral clusters. The implication of our finding is that the sex differences we observed for cocaine and dopamine effects on LMA were driven by behavioral cluster effects and not by biological sex.

There are some limitations of this study. One limitation is that the estrous phases of the females were not considered, and these may play a role in the behavioral clusters. While we do not believe that this is the case, it needs to be clarified. We plan to conduct a study in the future to investigate whether the behavioral clusters are defined by the estrous phase. A second limitation is that we used Sprague Dawley rats, which represent an outbred strain. This outbred strain implies that every individual has a slightly different genetic background. It is unclear whether these differences in genetic background affect the cluster classification. Therefore, it is necessary to examine the genetic profile of every individual using genetic markers throughout the genome. Future efforts should attempt to reproduce these results in inbred rat strains. A third limitation is that the rats were tested during their natural sleep phase, and this approach could significantly affect the validity of the findings, because the baseline and drug-induced LMA may be skewed by the altered activity typical of their sleep state and therefore may not accurately represent the rats’ true baseline LMA or LMA responses to psychostimulants. In the future, we plan to replicate our findings while accounting for the sleep phase of the rats.

### Conclusions

The MISSING model proposes that observed differences (in psychostimulant-induced LMA) between males and females may not always be related to biological sex but rather to a behavioral group identity that is independent of sex, which we describe for the first time. This work has significant implications for how we proceed with research aimed at understanding the mechanisms that govern sex differences in behavior.

## Supplementary Material

Supplemental Results, Discussion, Figures S1–S9, and Tables S1–S2

Key Resource Table
