# Supplementary material for: Lack of Sex Differences in Psychostimulant-Induced Locomotor Activity When Comparing Rats From the Same Behavioral Groups
Source: Biol Psychiatry Glob Open Sci. 2025 Apr 25;5(5):100519. doi: 10.1016/j.bpsgos.2025.100519 (PMC12221628; doi:10.1016/j.bpsgos.2025.100519)
Supplement: Supplemental Results, Discussion, Figures S1–S9, and Tables S1–S2 [file mmc1.pdf]

SUPPLEMENTARY INFORMATION

Lack of Sex Differences in Psychostimulant-Induced Locomotor Activity When Comparing Rats From the Same Behavioral Groups

Tigano and Job

Hypothesis: Our hypothesis was that there would be no sex differences in saline and psychostimulant drug-induced locomotor activity (LMA) when we compared males and females belonging to the same behavioral group. This hypothesis was developed, in part, from evidence in literature, summarized in Table S1.

Testing our hypothesis: We tested our hypothesis via the following specific Aims:

Aim 1: Determine if there are biological sex differences in locomotor activity (LMA) following administration of saline via intraperitoneal injection after also accounting for the behavioral group identity of male and female Sprague Dawley rats.

Aim 2: Determine if there are biological sex differences in locomotor activity (LMA) following administration of cocaine (10 mg/kg) via intraperitoneal injection after also accounting for the behavioral group identity of male and female Sprague Dawley rats.

Aim 3: Determine if there are biological sex differences in locomotor activity (LMA) following intra-nucleus accumbens (intra-NAc) administration of dopamine (15 µg/ 0.5 µL/ side) after also accounting for the behavioral group identity of male and female Sprague Dawley rats.

Behavioral Group Identification: A current method for identifying behavioral groups is to employ median split of drug-induced LMA. The challenge currently in the field is that the median split procedure is limited in its ability to identify behavioral groups (see Introduction in the main manuscript). To address the limitations of the median split, we developed the MISSING model which employs unbiased normal mixtures clustering of several variables (not one, as is done with the median split) to identify behavioral groups that are truly distinct. We defined our MISSING model, see Figure S1.

Model Comparison: We tested our hypothesis with the MISSING model and compared our results to the current model (median split).

Results

We defined our variables, including a novel baseline activity-time normalized variable (Figure 1, main manuscript).

Assessment of injection site coordinates for the intra-nucleus accumbens dopamine injected subjects

For the dopamine-induced LMA experiments, we directly injected dopamine into the NAc core. We compared the injection sites between males and females. Every subject had bilateral injection coordinates and as such for n = 20 males, we had 40 injection coordinates and for n = 17 females, we had 34 injection coordinates. For males (n = 40), the mean ± SEM for AP (anteroposterior), ML (mediolateral) and DV (dorsoventral) were 1.29 ± 0.07 mm, 1.53 ± 0.04 mm and 7.17 ± 0.07 mm, respectively. For males (n = 34), the mean ± SEM for AP, ML and DV were 1.29 ± 0.09 mm, 1.46 ± 0.03 mm and 7.36 ± 0.08 mm, respectively. The injection site coordinates for all subjects are shown in Figure S2. Comparisons of males and females using unpaired t-tests revealed no significant differences for AP (P = 0.9356, Figure S2B), ML (P = 0.3922, Figure S2C) and DV (P = 0.3014, Figure S2D) injection site coordinates.

LMA assessments

For Methodology regarding LMA assessments, see Method section in main manuscript. We compared several variables and detected sex differences in cocaine and dopamine-induced locomotor activity (LMA), as expected (Figure 2, main manuscript)

When we integrated all the variables for all subjects for saline, cocaine and dopamine, we determined that there were more than one population of subjects in our samples (Figure 3, main manuscript). Note that, when more than one population was detected, the median split procedure of the variable of interest was ineffective in distinguishing the populations revealed (see Figure 3, main manuscript).

Median split analysis

There were observed sex differences for cocaine and dopamine-induced LMA (Figure 2, main manuscript). However, when we divided our subjects (irrespective of biological sex) into high versus low responders (HR versus LR) for drug-induced LMA using the median split procedure, we determined that there was no SEX × group interaction. We grouped our subjects as LR-a versus HR-a and LR-b versus HR-b depending on whether we included the median value as the upper limit of the LR group or the lower limit of the HR group. For cocaine LR-a versus HR-a,

the SEX × group interaction revealed no significance: $F_{1, 41} = 3.915$ ($P = 0.0546$). For cocaine LR-b versus HR-b, there was no SEX × group interaction: $F_{1, 41} = 3.993$ ($P = 0.0523$). For dopamine LR-a versus HR-a, the SEX × group interaction was not significant ($F_{1, 33} = 0.2811$, $P = 0.5995$), and for dopamine LR-b versus HR-b also, we determined that there was no significant SEX × group interaction ($F_{1, 33} = 0.6273$, $P = 0.4340$). For all comparisons (cocaine, dopamine), there were main effects of group ($P < 0.0001$). For all comparisons (cocaine, dopamine), there were no main effects of SEX ($P > 0.05$). For plots of the data above, see Figure 4 (main manuscript).

Distinctions between behavioral groups identified via the median split procedure

As mentioned previously, depending on whether we included the median value as the upper limit of the LR group or the lower limit of the HR group, we identified LR-a versus HR-a and LR-b versus HR-b. We compared six variables (baseline LMA, drug-induced LMA, drug-induced LMA_nba, $ß_0$, $ß_1$ and $ß_2$, see variables in Figure 1) between LR-a versus HR-a (see Figure S3) and 2) between LR-b versus HR-b (see Figure S4).

LR-a versus HR-a

For saline, we identified differences (using unpaired t-tests) between these groups for baseline LMA, saline-induced LMA, saline-induced LMA_nba, $ß_0$, (Figure S3 A-D) but not for $ß_1$ and $ß_2$ (Figure S3 E-F). Thus, LR-a and HR-a (saline) were different for 4 out of 6 variables (or 66.7% of the variables assessed). For cocaine, we identified differences between these groups for baseline LMA and cocaine-induced LMA, but not for the other variables (groups were different for only 33.3% of the variables assessed). For dopamine, we identified differences between these groups for baseline LMA, dopamine-induced LMA, dopamine-induced LMA_nba and for $ß_1$ but not for $ß_0$ and $ß_2$ (Figure S3). Thus, LR-a and HR-a (dopamine) were different for 3 out of 6 variables (or 50% of the variables assessed). Open circles represent males while closed circles represent females. The clusters are represented by different colors. P values are written in the graphs. Comparisons, after significant differences were detected, were conducted using Tukey's post hoc tests.

LR-b versus HR-b

For saline, we identified differences (using unpaired t-tests) between these groups for baseline LMA, saline-induced LMA, $ß_0$, and $ß_2$ (Figure S4 A, B, D and F) but not for saline-induced LMA_nba and $ß_1$ (Figure S4 C and E). Thus, LR-a and HR-a (saline) were different for 4 out of 6 variables (or 66.7% of the variables assessed). For cocaine, we identified differences for only 33.3% of the variables assessed. For dopamine, we identified differences (using unpaired t-tests) between these groups for dopamine-induced LMA, dopamine-induced LMA_nba and for $ß_1$ but not for baseline LMA, $ß_0$ and $ß_2$ (see Figure S4). Thus, LR-a and HR-a (dopamine) were different for 3 out of 6 variables (or 50% of the variables assessed). Open circles represent males while closed circles represent females. The clusters are represented by different colors. P values are written in the graphs. Comparisons, after significant differences were detected, were conducted using Tukey's post hoc tests.

Subjectivity of the median split procedure

Note that LR-a versus HR-a were more different than LR-b versus HR-b for saline-induced LMA_nba (Figure S3C versus Figure S4C). Conversely, LR-a versus HR-a were less different than LR-b versus HR-b for ß2 (Figure S3F versus Figure S4F)– the variable that represents the slope of the relationship between drug-induced LMA and drug-induced LMA_nba. *This shows that the median split procedure is not without subjectivity, the group compositions can vary depending on where the experimenter places the median value (as the upper limit of the LR or the lower limit of the HR).*

The MISSING model

Saline-induced LMA

Normal mixtures clustering of baseline LMA, saline-induced LMA and saline-induced LMA_nba of all subjects (N = 23) revealed 2 clusters each consisting of males and females (Figure S5A-B). We labeled these as cluster1 (N = 12: males n = 7, females n = 5) and cluster2 (N = 11: males n = 5, females n = 6). The values of $ß_0$, $ß_1$ and $ß_2$ were obtained for each cluster from multiple regression analysis on a 3-D plane using equation $Z = ß_0 + ß_1X + ß_2Y$ (see Figure 1, main manuscript for variable definitions). Unpaired t-tests revealed significant differences between cluster1 and cluster2 for $ß_0$ (intercept of the behavioral interaction complex on the Z

axis) ($ß_0$, $P < 0.0001$, Figure S5F), slope of X-Z axis ($ß_1$, $P < 0.0001$, Figure S5G) but not for slope on the Y-Z axis ($ß_2$, $P = 0.6032$, Figure S5H). The values obtained for each cluster with respect to baseline LMA, saline-induced LMA and saline-induced LMA_nba are shown in Table 2 (main manuscript).

Unpaired t-tests revealed no significant differences between cluster1 and cluster2 for baseline LMA ($P = 0.8886$, Figure S5I) and saline-induced LMA ($P = 0.0513$, Figure S5J), but revealed significant differences for saline-induced LMA_nba ($P < 0.0001$, Figure S5K). The slopes of the relationship between baseline LMA and saline-induced LMA (Figure S5L) were significant for both clusters: cluster1 (F 1, 10 = 31.16, $P = 0.0002$, $R^2 = 0.76$, slope = 1.426 ± 0.2555), cluster2 (F 1, 9 = 277, $P < 0.0001$, $R^2 = 0.97$, slope = 2.633 ± 0.1582). For the relationship between baseline LMA and saline-induced LMA, linear regression analysis revealed that there were significant differences between cluster1 and cluster2 for slope (F 1, 19 = 16.37, $P = 0.0007$, Figure S5L). For the relationship between baseline LMA and saline-induced LMA_nba, linear regression analysis revealed no significant differences (cluster1 versus cluster2) between slopes (F 1, 19 = 0.001101, $P = 0.9739$, Figure S5M). For the relationship between saline-induced LMA and saline-induced LMA_nba, linear regression analysis revealed that there were no differences between clusters for slope (F 1, 19 = 1.632, $P = 0.2169$).

We wanted to know if there were sex differences for baseline LMA, saline-induced LMA and saline-induced LMA_nba, when we account for the clusters. For this, we employed a Two-way ANOVA with factors SEX (males and females) and cluster (cluster1, cluster2). We did not detect any SEX × cluster interactions. The statistics are shown in Table S2.

MISSING model versus median split: group composition

The broken lines in Figure S5A represent median split of baseline and saline-induced LMA – median split distinguished two groups that were dissimilar to the clusters. The groups identified via median split of saline-induced LMA represented combinations of individuals from different clusters (Figure S5C).

Cocaine-induced LMA

Normal mixtures clustering of baseline LMA, cocaine-induced LMA and cocaine-induced LMA_nba revealed 3 clusters, with all clusters consisting of males and females (Figure S6A-B).

We labeled these as cluster1 (N = 22: males n = 12, females n = 10), cluster2 (N = 14: males n = 7, females n = 7) and cluster3 (N = 9: males n = 3, females n = 6). One-way ANOVA revealed significant differences between clusters 1-3 for all 3-D plane variables: $ß_0$: F 2, 42 = 61.09, P < 0.0001 (Figure S6G), $ß_1$: F 2, 42 = 47.51, P < 0.0001 (Figure S6H) and $ß_2$: F 2, 42 = 3.818, P = 0.0300 (Figure S6I). The values obtained for baseline LMA, cocaine-induced LMA and cocaine-induced LMA_nba are shown in Table 2.

One-way ANOVA revealed significant differences between clusters for baseline LMA (F 2, 24 = 20.34, P < 0.0001, Figure S6J) and cocaine-induced LMA_nba (F 2, 24 = 82.36, P < 0.0001, Figure S6L), but not for cocaine-induced LMA (F 2, 24 = 2.012, P = 0.1464, Figure S6K). The slopes of the relationship between baseline LMA and cocaine-induced LMA (Figure S6M) were significant for all clusters: cluster1 (F 1, 20 = 47.76, P < 0.0001, R^2 = 0.71, slope = 13.06 ± 1.89), cluster2 (F 1, 12 = 34.34, P < 0.0001, R^2 = 0.74, slope = 11.72 ± 2.00) and cluster3 (F 1, 7 = 7.064, P = 0.0326, R^2 = 0.50, slope = 19.14 ± 7.203). However, we observed that there were no differences between clusters with respect to slopes (F 2, 39 = 0.5006, P = 0.6100). Similarly (Figure S6N), the slopes of the relationship between baseline LMA and cocaine-induced LMA_nba were significant for all clusters: cluster3 (F 1, 7 = 11.48, P = 0.0116, R^2 = 0.62, slope = -0.007086 ± 0.002092), cluster2 (F 1, 12 = 11.10, P = 0.0060, R^2 = 0.48, slope = -0.0009442 ± 0.0002834) and cluster1 (F 1, 20 = 7.497, P = 0.0127, R^2 = 0.27, slope = +0.0004630 ± 0.0001691), and there were significant differences when we compared slopes for clusters 1-3 (F 2, 39 = 26.15, P < 0.0001). Interestingly, for the relationship between cocaine-induced LMA and cocaine-induced LMA_nba (Figure S6O), linear regression analysis revealed that there were no differences between clusters for slope (F 2, 39 = 2.477, P = 0.0971).

We wanted to know if there were sex differences for baseline LMA, cocaine-induced LMA and cocaine-induced LMA_nba, when we account for the clusters. For this, we employed a Two-way ANOVA with factors SEX (males and females) and cluster (cluster1, cluster2 and cluster3). We did not detect any SEX × cluster interactions. The statistics are shown in Table S2.

MISSING model versus median split: group composition

The broken lines in Figure S6A represent median split of baseline and cocaine-induced LMA – median split distinguished two groups that were dissimilar to the clusters. The groups identified

via median split of cocaine-induced LMA represented combinations of individuals from different clusters (Figure S6C).

Dopamine-induced LMA

Normal mixtures clustering of baseline LMA, dopamine-induced LMA and dopamine-induced LMA_nba revealed 3 clusters, with two clusters consisting of males and females, and one cluster consisting of only females (Figure S7A-B). We labeled these as cluster1 (N = 23: males n = 16, females n = 7), cluster2 (n = 11: males n = 4, females n = 7) and cluster3 (N = 3: males n = 0, females n = 3). For each cluster, the values for $ß_0$, $ß_1$ and $ß_2$ were obtained from multiple linear regression analysis- the XYZ plots are shown (Figure S7D-F) and compared (Figure S7G-I). The values obtained for baseline LMA, dopamine-induced LMA and dopamine-induced LMA_nba are shown in Table 2.

One-way ANOVA revealed no significant differences between clusters for baseline LMA (F 2, 34 = 2.237, P = 0.1223, Figure S7J), but significant differences between clusters with regards to dopamine-induced LMA (F 2, 34 = 64.04, P < 0.0001, Figure S7K) and dopamine-induced LMA_nba (F 2, 34 = 60.87, P < 0.0001, Figure S7L). The F values, P values and goodness of fit (R^2) for the lines representing the relationship between baseline LMA and dopamine-induced LMA (Figure S7M) were as follows: cluster1 (F 1, 21 = 20.29, P = 0.0002, R^2 = 0.49), cluster2 (F 1, 9 = 2.637, P = 0.1389, R^2 = 0.23) and cluster3 (F 1, 1 = 73.61, P = 0.0739, R^2 = 0.99). For Figure S7M, there were no differences between clusters with respect to slopes (F 2, 31 = 1.430, P = 0.2545). For the lines representing the relationship between baseline LMA and dopamine-induced LMA_nba (Figure S7N), the F values, P values and goodness of fit (R^2) were as follows: cluster1 (F 1, 21 = 6.742, P = 0.0168, R^2 = 0.24), cluster2 (F 1, 9 = 7.872, P = 0.0205, R^2 = 0.47) and cluster3 (F 1, 1 = 10.05, P = 0.1945, R^2 = 0.91). For Figure S7N, we detected significant differences between clusters with respect to the slopes (F 2, 31 = 76.53, P < 0.0001). The differences between clusters could not be explained by dopamine injection site coordinates. One way ANOVA revealed the following results: AP (F 2, 34 = 0.5621, P = 0.5752), ML (F 2, 34 = 0.8245, P = 0.4470 and DV (F 2, 34 = 0.1176, P = 0.8894). Additionally, for the relationship between dopamine-induced LMA and dopamine-induced LMA_nba (Figure S7O), linear regression analysis revealed significant differences between clusters for slope (F 2, 31 = 80.96, P < 0.0001).

Having obtained the clusters, we explored SEX × cluster interaction using Two-way ANOVA with factors SEX (males, females) and clusters (cluster1-2). *We excluded cluster 3 from this specific analysis because it consisted of only females* (see Figure S7A-B). For baseline-induced LMA, Two-way ANOVA did not reveal a SEX × cluster interaction ($F_{1, 30} = 0.3294$, $P = 0.5703$) or a main effect of cluster ($F_{1, 30} = 0.5182$, $P = 0.4772$) but did reveal a main effect of SEX ($F_{1, 30} = 6.676$, $P = 0.0149$). For dopamine-induced LMA, Two-way ANOVA did not reveal a SEX × cluster interaction ($F_{1, 30} = 2.051$, $P = 0.6539$) or a main effect of SEX ($F_{1, 30} = 2.690$, $P = 0.1114$) but did reveal a main effect of cluster ($F_{1, 30} = 82.50$, $P < 0.0001$). Similarly, for dopamine-induced LMA_nba, Two-way ANOVA did not reveal a SEX × cluster interaction ($F_{1, 30} = 2.973$, $P = 0.0949$) or a main effect of SEX ($F_{1, 30} = 3.450$, $P = 0.0731$) but did reveal a main effect of cluster ($F_{1, 30} = 89.72$, $P < 0.0001$).

No sex differences within a behavioral cluster (dopamine)

Comparisons between males and females in cluster1, using unpaired t-tests, revealed significant differences for baseline LMA ($P = 0.0208$, Figure S8A), but no significant differences for dopamine-induced LMA ($P = 0.0826$, Figure S8B) and dopamine-induced LMA_nba ($P = 0.8689$, Figure S8C). Comparisons between males and females in cluster2 revealed no significant differences for baseline LMA ($P = 0.1504$, Figure S8D), dopamine-induced LMA ($P = 0.4449$, Figure S8E) and dopamine-induced LMA_nba ($P = 0.1752$, Figure S8F). Interestingly, comparisons between males from cluster1 and females from cluster2 revealed no significant differences for baseline LMA ($P = 0.0846$, Figure S8G), but significant differences for dopamine-induced LMA ($P < 0.0001$, Figure S8H) and dopamine-induced LMA_nba ($P < 0.0001$, Figure S8I). Likewise, comparisons between males from cluster2 and females from cluster1 revealed no significant differences for baseline LMA ($P = 0.1340$, Figure S8J), but significant differences for dopamine-induced LMA ($P = 0.0003$, Figure S8K) and dopamine-induced LMA_nba ($P < 0.0001$, Figure S8L). Thus, sex differences in dopamine-related effects appear to be driven by cluster differences not biological sex *per se*: for all variables (Figure S8), there were no sex differences *within* any cluster but there were sex differences when we compared males and females from different clusters.

The most significant sex differences are realized when we compare males and females from different behavioral clusters (dopamine)

While there were no males in cluster3 (Figure S7A-B), comparisons between females in cluster3 and males in cluster1 revealed no differences for baseline LMA ($P = 0.0924$, Figure S9A), but differences for dopamine-induced LMA ($P < 0.0001$, Figure S9B) and dopamine-induced LMA_nba ($P < 0.0001$, Figure S9C). Comparisons between females in cluster3 and males in cluster2 revealed no differences for baseline LMA ($P = 0.1194$, Figure S9D) and for dopamine-induced LMA ($P = 0.2713$, Figure S9E), but differences for dopamine-induced LMA_nba ($P = 0.0403$, Figure S9F). Comparisons between females in cluster3 and females in cluster1 revealed differences for all variables: baseline LMA ($P = 0.0331$, Figure S9G), dopamine-induced LMA ($P < 0.0001$, Figure S9H) and dopamine-induced LMA_nba ($P = 0.0010$, Figure S9I). Comparisons between females in cluster3 and females in cluster2 revealed differences for baseline LMA ($P = 0.0085$, Figure S9J) and for dopamine-induced LMA_nba ($P = 0.0033$, Figure S9L), but no differences for dopamine-induced LMA ($P = 0.9371$, Figure S9K). Nore that the females in cluster3 were significantly different for 3/3 variables when compared to females in cluster1 (Figure S9G, H and I), but different for only 1/3 variables when compared to males in cluster 2 (Figure S9D, E and F). *This implies that females in cluster3 were more different (behaviorally) from females in cluster1 than they were from males in cluster2.* Females in cluster3 were as different from males in cluster1 (Figure S9B) as they were different from females in cluster1 (Figure S9H). As such the difference between females in cluster3 and males in cluster1 are not due to biological sex-identity, but rather due to behavioral group-identity.

Discussion

From careful analysis of the literature (Table S1), we hypothesized that there would be no sex differences in psychostimulant (cocaine or dopamine)-induced LMA when we compare males and females from the same behavioral groups. Because of the limitations saddling the current grouping strategy (median split) in identifying distinct behavioral groups, we developed a new model termed the MISSING model (Figure S1). The MISSING model proposes that, for psychostimulant-induced LMA, 1) there are no sex differences when we compare males and females within the same behavioral cluster, 2) sex differences will be observed mostly when we

compare males and females from different behavioral clusters, and 3) even if/when we detect sex differences between males and females in the same behavioral cluster, these differences will not be as significant as differences between males and females from different clusters. Our aim was to test our hypothesis using the MISSING model.

When we compared males and females, we determined that there were differences in cocaine-induced LMA and dopamine-induced LMA, as expected (Figure 2). Interestingly, we determined that there were no sex differences in a baseline activity-time normalized LMA variable (Figure 2). We detected more than one population of males and females in our sample based on distribution analysis and the integration/cluster analysis of several variables (Figure 3, 5). We realized that despite observing differences when subjects were grouped by biological sex (Figure 2), there were no longer any differences when subjects were (also) grouped via the median split (Figure 4) or via normal clustering analysis (Figure 5). There were always observed main effect(s) of behavioral group(s) (Figure 4, Table S2). There were no main effect(s) of biological sex when we accounted for behavioral group-identity (Table S2, see also Figure S8-9).

We confirmed that normal mixtures clustering did not reveal the same groups identified via median split (Figure 5, Figure S5-7). Normal mixtures clustering analysis identified more distinct groups and represented a more effective grouping strategy than the median split procedure (Figure 6 versus Figure S3-4, see also Figure S5-7).

Regardless of grouping strategy (median split versus normal mixtures clustering), there were no main effects of sex but there were significant main effect(s) of group. Furthermore, the most significant differences between males and females were observed when males and females from different behavioral clusters were compared (Figure S8-9). The implication is that the differences between males and females that were observed were actually due to behavioral group identity rather than biological sex identity.

Our study validates the MISSING model.

References

1. Davis BA, Clinton SM, Akil H, Becker JB (2008): The effects of novelty-seeking phenotypes and sex differences on acquisition of cocaine self-administration in selectively bred High-Responder and Low-Responder rats. *Pharmacol Biochem Behav* 90: 331–338.

2. Brown JD, Green CL, Arthur IM, Booth FW, Miller DK (2015): Cocaine-induced locomotor activity in rats selectively bred for low and high voluntary running behavior. *Psychopharmacology (Berl)* 232: 673–681.

3. Carroll ME, Anderson MM, Morgan AD (2007): Higher locomotor response to cocaine in female (vs. male) rats selectively bred for high (HiS) and low (LoS) saccharin intake. *Pharmacol Biochem Behav* 88: 94–104.

4. Carreira MB, Cossio R, Britton GB (2017): Individual and sex differences in high and low responder phenotypes. *Behav Processes* 136: 20–27.

5. Paxinos G, Watson C (1998): *The Rat Brain in Stereotaxic Coordinates.*, 4th ed. Burlington MA: Academic Press Inc.

Table S1: The observation of sex differences or sex similarities may depend on the behavioral groups of males and females that are compared.

strain	RAT	RAT	RAT	MICE
SEX	Males v females	Males v females	Males v females	Males v females
Behavioral group(s)	High responder (HR) versus low responder (LR)	High voluntary running (HVR) versus low voluntary running (LVR)	High saccharin consumption (HSC) versus low saccharin consumption (LSC)	High responder (HR) versus low responder (LR)
Baseline LMA (LMA in a novel environment)	Sex differences for LR males versus LR females<br><br>No sex differences for HR males versus HR females	No sex differences when males and females within the same behavioral group were compared		No sex differences when males and females within the same behavioral group were compared
Saline-induced LMA			Sex differences for HSC males versus LSC females<br><br>No sex differences for LSC males versus LSC females	
Cocaine-induced LMA (after first cocaine injection)			Sex differences for HSC males versus LSC females<br><br>No sex differences for LSC males versus LSC females	
Reference	(1)	(2)	(3)	(4)

Table S2: Statistics showing the analysis of the baseline LMA, drug-induced LMA and drug-induced LMA_nba for saline and cocaine. We employed Two-way ANOVA with SEX (males, females) and Cluster (Clusters 1, 2 or more). We did not detect any SEX × cluster interactions. We detected a main effect of cluster for saline and cocaine-induced LMA_nba. We did not detect a main effect of SEX. The * show significant difference (P < 0.05).

Drug	condition	SEX × cluster interaction	Cluster (main effect)	SEX (main effect)
Saline	Baseline LMA	($F_{1, 19}$ = 0.4894, P = 0.4927)	($F_{1, 19}$ = 0.03309, P = 0.8576)	($F_{1, 19}$ = 0.3128, P = 0.5825)
	Saline-induced LMA	($F_{1, 19}$ = 0.7203, P = 0.4066)	($F_{1, 19}$ = 3.748, P = 0.0679)	($F_{1, 19}$ = 0.9547, P = 0.3408)
	Saline-induced LMA_nba	($F_{1, 19}$ = 0.3291, P = 0.5729)	($F_{1, 19}$ = 80.34, P < 0.0001) (*)	($F_{1, 19}$ = 2.196, P = 0.1548)
Cocaine	Baseline LMA	($F_{2, 39}$ = 0.4764, P = 0.6244)	($F_{2, 39}$ = 22.70, P < 0.0001) (*)	($F_{1, 39}$ = 3.512, P = 0.0684)
	Cocaine-induced LMA	($F_{2, 39}$ = 0.4833, P = 0.6204)	($F_{2, 39}$ = 1.562, P = 0.2225)	($F_{1, 39}$ = 2.948, P = 0.0939)
	Cocaine-induced LMA_nba	($F_{2, 39}$ = 2.605, P = 0.0867)	($F_{2, 39}$ = 87.50, P < 0.0001) (*)	($F_{1, 39}$ = 1.957, P = 0.1697)

Figure Legends

Figure S1: Sex differences as an overestimation of distinctions between males and females in different, not the same, behavioral groups: the MISSING model. Fig A represents the current model wherein, prior to comparisons, males and females are assumed to represent distinct behavioral groups based on biological sex. Fig B is the MISSING (Mapping Intrinsic Sex Similarities as an Integral quality of Normalized Groups) model which does not assume that males and females represent distinct behavioral groups prior to comparisons, instead it conducts normal mixtures clustering of all individuals, regardless of sex, to identify the behavioral groups they belong to before assessing the impact of sex on behavior. The square in Fig B captures two clusters (cluster1 and 2) consisting of both males and females. The thick and thin lines around the square represent differences and similarities, respectively. The MISSING model proposes that 1) there are no sex differences when we compare males and females within the same behavioral group, 2) sex differences are observed when we compare males and females from different groups, and 3) even if/when we detect sex differences between males and females in the same behavioral group, these differences will not be as significant as differences between males and females from different groups. Sex differences in the current model (Fig A) are likely due to comparisons of males and females from different clusters as in Fig B. The goal of this study was to test this model.

Figure S2: Histology showing placement of injection points bilaterally in male and female Sprague Dawley rats. The brain drawings are from (5) with injection coordinates from bregma (mm). The red triangles represent females while the blue squares represent males (Fig A). We employed unpaired t-tests for a comparison of the injection placements in the anterior-posterior (AP, Fig B), medial-lateral (ML, Fig C) and dorsal-ventral (DV, Fig D) axis of the nucleus accumbens core. For these males and females are shown as blue open squares and red closed circles, respectively. We determined that there were no sex differences with regards to injection tip placements (bilaterally).

Figure S3: Comparing several variables for groups identified via median split of drug-induced LMA: the median was placed in the upper limit of the LR group. For LMA assessments following saline (top row), cocaine (mid row) and dopamine (bottom row), we conducted median split of drug-induced LMA (B, H and N). For the groups derived we estimated

the values of baseline LMA (A, G and M), drug-induced LMA_nba (C, I and O), $ß_0$ (D, J and P), $ß_1$ (E, K and Q) and, $ß_2$ (F, L and R) and compared LR versus HR using unpaired t-tests. The P values are written in the graphs. We expressed the number of variables different/ total number of variables as % differences. The saline groups were different for 4 out of 6 variables or 66.7% whereas the cocaine groups were different for 2 out of 6 variables (or 33.3%). The saline groups appeared to be the most distinct whereas the cocaine groups appeared to be the least distinct. The dopamine groups were distinct for 50% of the variables.

Figure S4: Comparing several variables for groups identified via median split of drug-induced LMA: the median was placed in the lower limit of the HR group. For LMA assessments following saline (top row), cocaine (mid row) and dopamine (bottom row), we conducted median split of drug-induced LMA (B, H and N). For the groups derived we estimated the values of baseline LMA (A, G and M), drug-induced LMA_nba (C, I and O), $ß_0$ (D, J and P), $ß_1$ (E, K and Q) and, $ß_2$ (F, L and R) and compared LR versus HR using unpaired t-tests. The P values are written in the graphs. We expressed the number of variables different/ total number of variables as % differences. The saline groups were different for 4 out of 6 variables or 66.7% whereas the cocaine groups were different for 2 out of 6 variables (or 33.3%). The saline groups appeared to be the most distinct whereas the cocaine groups appeared to be the least distinct. The dopamine groups were distinct for 50% of the variables.

Figure S5: Sex differences/similarities for baseline LMA, saline-induced LMA and saline-induced LMA_nba when variables are analyzed together. We conducted normal mixtures clustering of all these 3 variables for all subjects (N = 23). This analysis yielded two clusters each consisting of males and females (A-B). Median split revealed groups that included members from each of these clusters (Fig C). We conducted multiple linear regression analysis and derived a 3-D plot of baseline LMA, saline-induced LMA and saline-induced LMA_nba (D-E) to yield variables $ß_0$, $ß_1$ and $ß_2$ (F-H). These clusters were distinct with regards to the variables $ß_0$ (F) and $ß_1$ (G), but not $ß_2$ (H). These clusters were distinct with regards to the normalized variable (K) but not baseline LMA and saline-induced LMA (I-J). These clusters were distinct with regards to the slope of the relationship between baseline LMA and saline-induced LMA (L) but not with saline-induced LMA_nba (M). The clusters were not distinct with regards to the slope of the relationship between saline-induced LMA and saline-induced LMA_nba (N). The *

show significant difference ($P < 0.05$). The analysis of the impact of these clusters on sex differences in baseline LMA, saline-induced LMA and saline-induced LMA_nba are shown in Table S2.

Figure S6: Sex differences/similarities for baseline LMA, cocaine-induced LMA and cocaine-induced LMA_nba when variables are analyzed together. We conducted normal mixtures clustering of all these 3 variables for all subjects (N = 45). This analysis yielded three clusters each consisting of males and females (A-B). Median split revealed groups that included members from each of these clusters (Fig C). We conducted multiple linear regression analysis and derived a 3-D plot of baseline LMA, cocaine-induced LMA and cocaine-induced LMA_nba (D-F) to yield variables $ß_0$, $ß_1$ and $ß_2$ (G-I). These clusters were distinct with regards to all these variables (G-I). The identified clusters were distinct with regards to the normalized variable (L) and baseline LMA (J), but not cocaine-induced LMA (K). These clusters were distinct with regards to the slope of the relationship between baseline LMA and cocaine-induced LMA_nba (N) but not for baseline versus cocaine-induced LMA (M) and cocaine-induced LMA v cocaine-induced LMA_nba (O). The * show significant difference ($P < 0.05$). The analysis of the impact of these clusters on sex differences in baseline LMA, cocaine-induced LMA and cocaine-induced LMA_nba are shown in Table S2.

Figure S7: Sex differences/similarities for baseline LMA, dopamine-induced LMA and dopamine-induced LMA_nba when variables are analyzed together. We conducted normal mixtures clustering of all these 3 variables for all subjects (N = 37). This analysis yielded three clusters each consisting of males and females (A-B) but with one cluster consisting of only females (cluster3). Groups identified via median split of dopamine-induced LMA are composed of subjects from different clusters (C). We conducted multiple linear regression analysis and derived a 3-D plot of baseline LMA, dopamine-induced LMA and dopamine-induced LMA_nba (D-F) to yield variables $ß_0$, $ß_1$ and $ß_2$ (G-I). We determined that the identified clusters were distinct with regards to the normalized variable (L) and dopamine-induced LMA (K) but not baseline LMA (J). These clusters were distinct with regards to the slope of the relationship between baseline LMA and dopamine-induced LMA_nba (N), dopamine-induced LMA and dopamine-induced LMA_nba (O), but not baseline LMA and dopamine-induced LMA (M). The * show significant difference ($P < 0.05$). Because there were no males in cluster3, we excluded it

when we wanted to determine if there was a SEX × cluster interaction (data in main text of manuscript). We, however, compared these females in cluster3 with males and females in clusters1-2, see Figure S9.

Figure S8. Behavioral cluster-matched and -nonmatched comparisons of males and females. We detected significant differences between cluster 1 males and females for baseline LMA (A), but no significant differences for dopamine-induced LMA (B), and dopamine-induced LMA_nba (C). We detected no significant differences between cluster 2 males and females for baseline LMA (D), dopamine-induced LMA (E), and dopamine-induced LMA_nba (F). When we conducted mismatched comparisons (cluster1 males versus cluster2 females, G-I OR cluster 2 males versus cluster1 females, J-L), we detected sex differences for dopamine-induced LMA (H, K) and dopamine-induced LMA_nba (I, L) but not for baseline LMA (G, J). In summary, sex differences are most pronounced when we conduct mismatched comparisons. As such these differences are likely not related to biological sex but to behavioral group/cluster identity.

Figure S9: Behavioral cluster differences exceed biological sex differences. Using unpaired t-tests, we compared the variables (baseline LMA, dopamine-induced LMA and dopamine-induced LMA_nba between females in cluster3 (nonmatched comparisons) and 1) males in cluster1 (A-C), 2) males in cluster2 (D-F), 3) females in cluster1 (G-I), and 4) females in cluster2 (J-L) A-C show no significant differences in baseline-induced LMA, but significant differences in dopamine-induced LMA and dopamine-induced LMA_nba for comparisons between males in cluster1 and females in cluster3. D-F reveal no significant differences in baseline LMA and dopamine-induced LMA, but significant differences for dopamine-induced LMA_nba for comparisons between males in cluster2 and females in cluster3. G-I reveal significant differences in baseline LMA, dopamine-induced LMA and dopamine-induced LMA_nba for comparisons between females from cluster1 and females from cluster3. J-L show significant differences in baseline LMA and dopamine-induced LMA_nba, but no significant differences in dopamine-induced LMA for comparisons between females from cluster2 and females from cluster3. Note that females in cluster3 are more similar to males in cluster2 (D-F) than they are to females in cluster1 (G-I). In summary, the most significant differences between sexes is driven by mismatched comparisons. Thus, the most significant differences between males and females

regarding dopamine-induced effects are driven by differences in behavioral cluster identity and not biological sex identity.

Figures

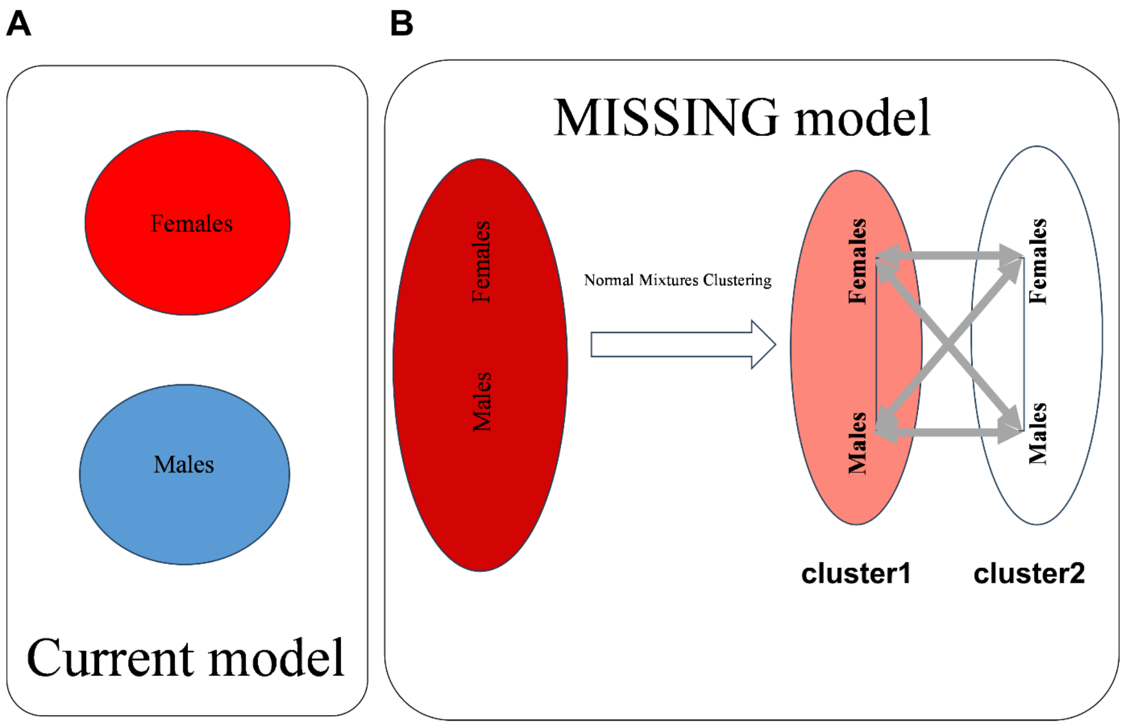

Figure S1

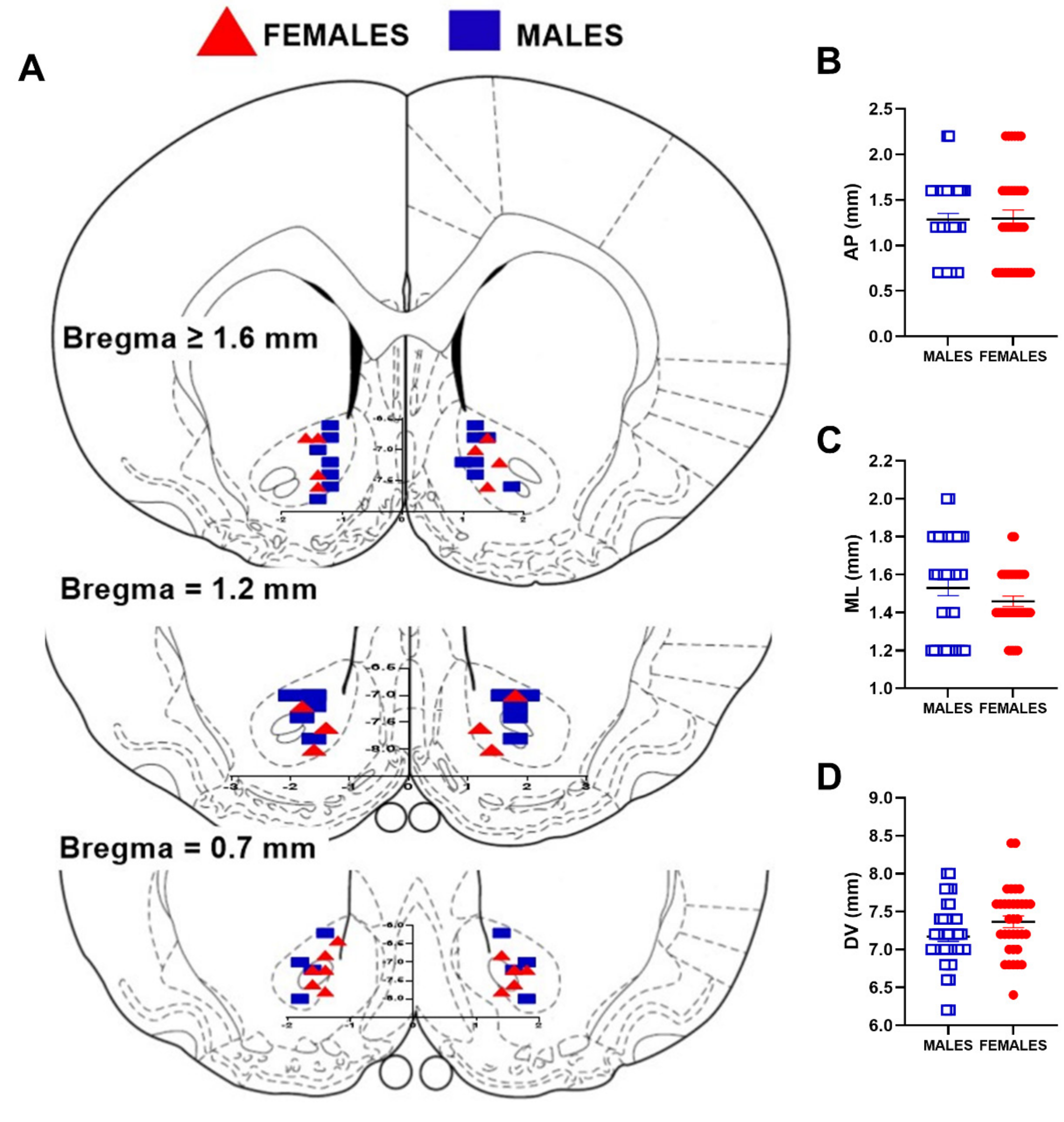
FEMALES
MALES
A
Bregma ≥ 1.6 mm
Bregma = 1.2 mm
Bregma = 0.7 mm
B
AP (mm)
MALES FEMALES
C
ML (mm)
MALES FEMALES
D
DV (mm)
MALES FEMALES

Figure S2

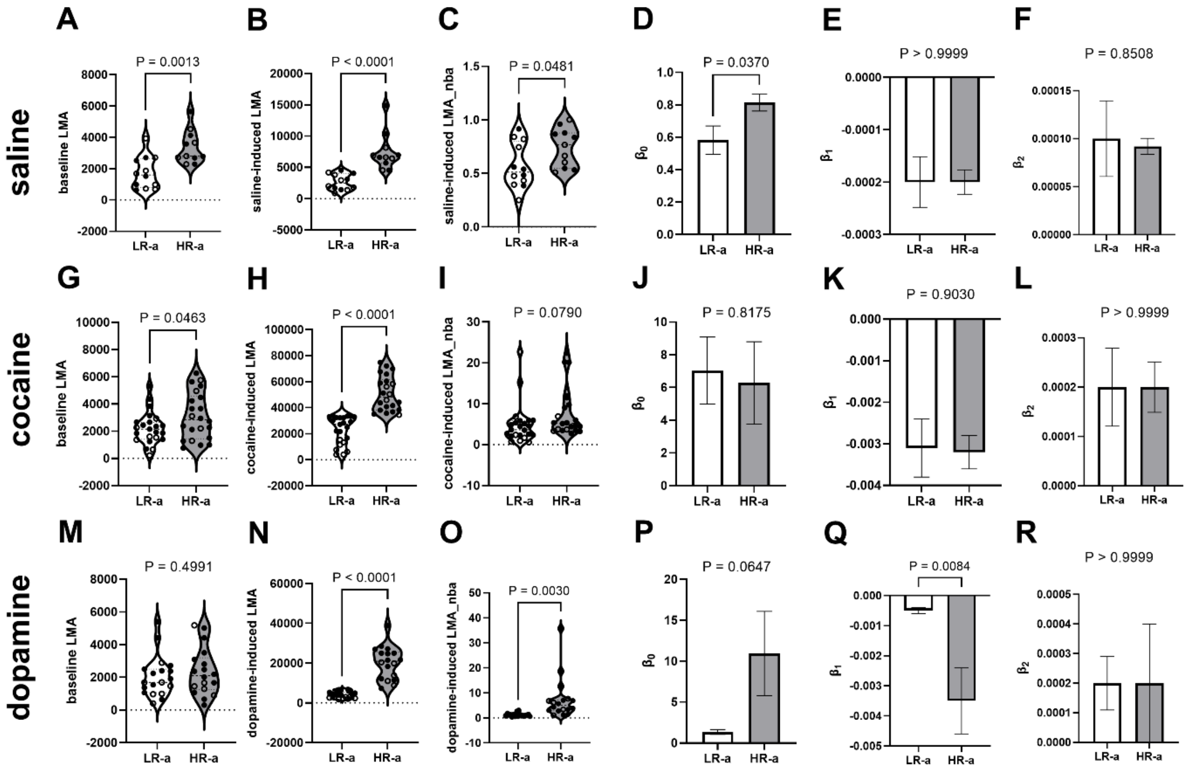

A
B
C
D
E
F
saline
P = 0.0013
P < 0.0001
P = 0.0481
P = 0.0370
P > 0.9999
P = 0.8508
baseline LMA
saline-induced LMA
saline-induced LMA_nba
LR-a
HR-a
G
H
I
J
K
L
cocaine
P = 0.0463
P < 0.0001
P = 0.0790
P = 0.8175
P = 0.9030
P > 0.9999
cocaine-induced LMA
cocaine-induced LMA_nba
M
N
O
P
Q
R
dopamine
P = 0.4991
P < 0.0001
P = 0.0030
P = 0.0647
P = 0.0084
P > 0.9999
dopamine-induced LMA
dopamine-induced LMA_nba

Figure S3

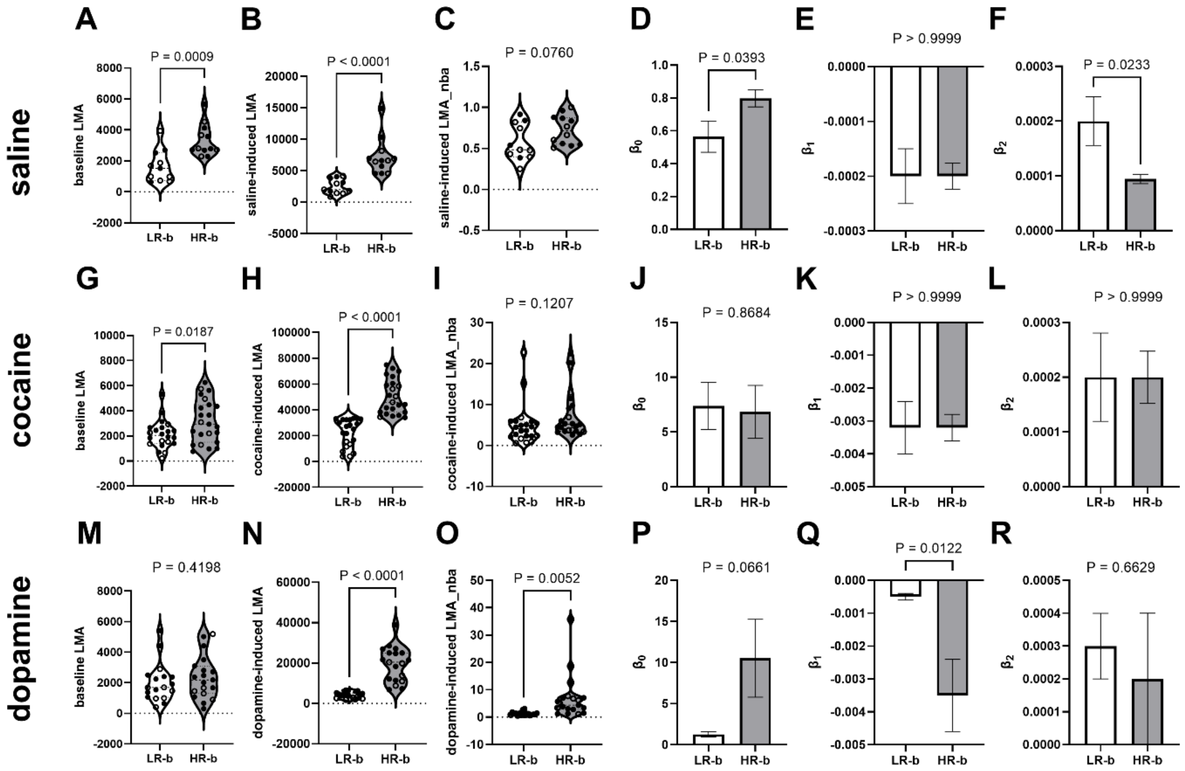
A
B
C
D
E
F
saline
P = 0.0009
P < 0.0001
P = 0.0760
P = 0.0393
P > 0.9999
P = 0.0233
baseline LMA
saline-induced LMA
saline-induced LMA_nba
LR-b
HR-b
G
H
I
J
K
L
cocaine
P = 0.0187
P < 0.0001
P = 0.1207
P = 0.8684
P > 0.9999
P > 0.9999
cocaine-induced LMA
cocaine-induced LMA_nba
M
N
O
P
Q
R
dopamine
P = 0.4198
P < 0.0001
P = 0.0052
P = 0.0661
P = 0.0122
P = 0.6629
dopamine-induced LMA
dopamine-induced LMA_nba

Figure S4

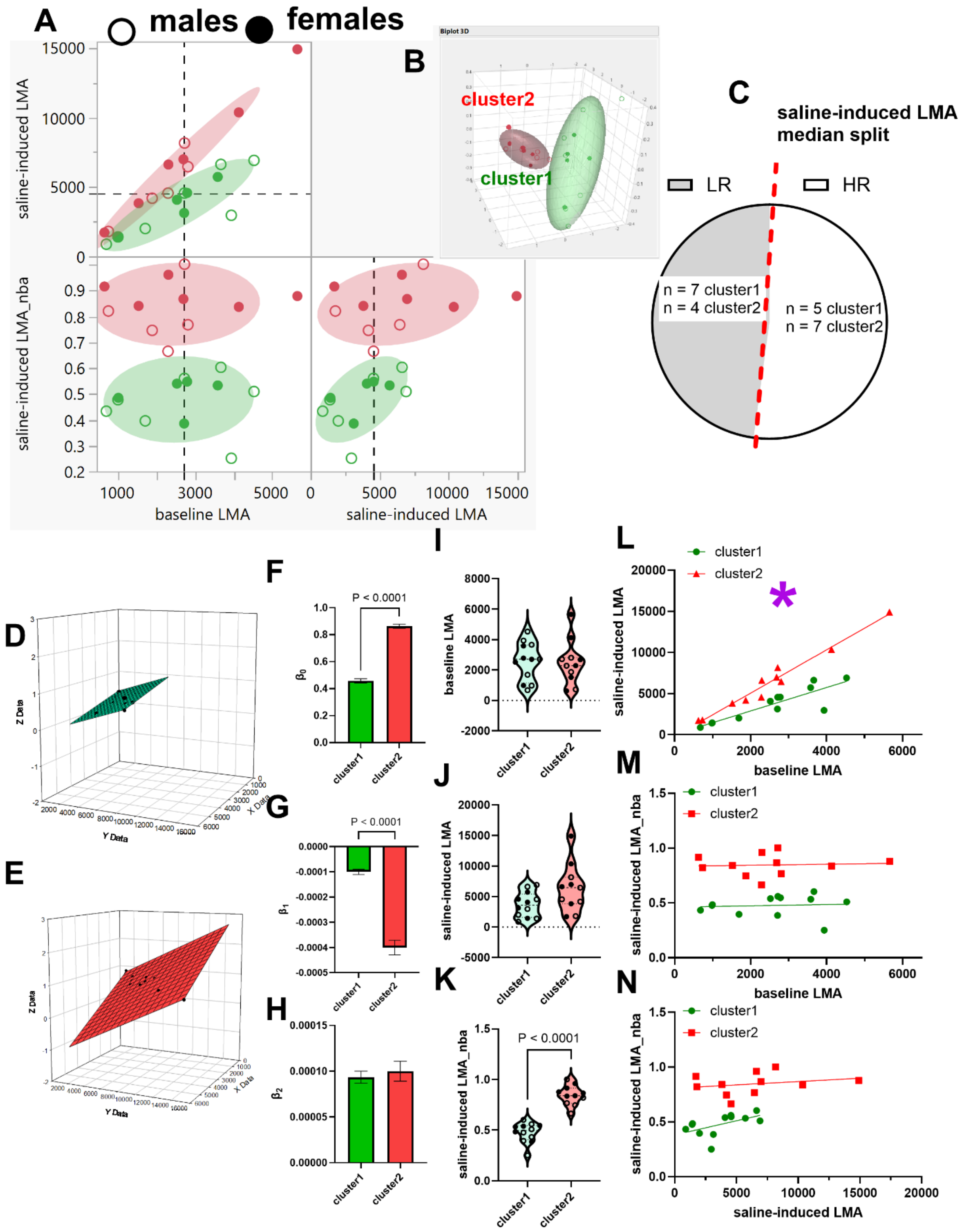
A
males
females
B
cluster2
cluster1
C
saline-induced LMA median split
LR
HR
n = 7 cluster1
n = 4 cluster2
n = 5 cluster1
n = 7 cluster2
saline-induced LMA
saline-induced LMA_nba
baseline LMA
D
E
F
G
H
I
J
K
L
M
N
P < 0.0001
cluster1
cluster2
*

Figure S5

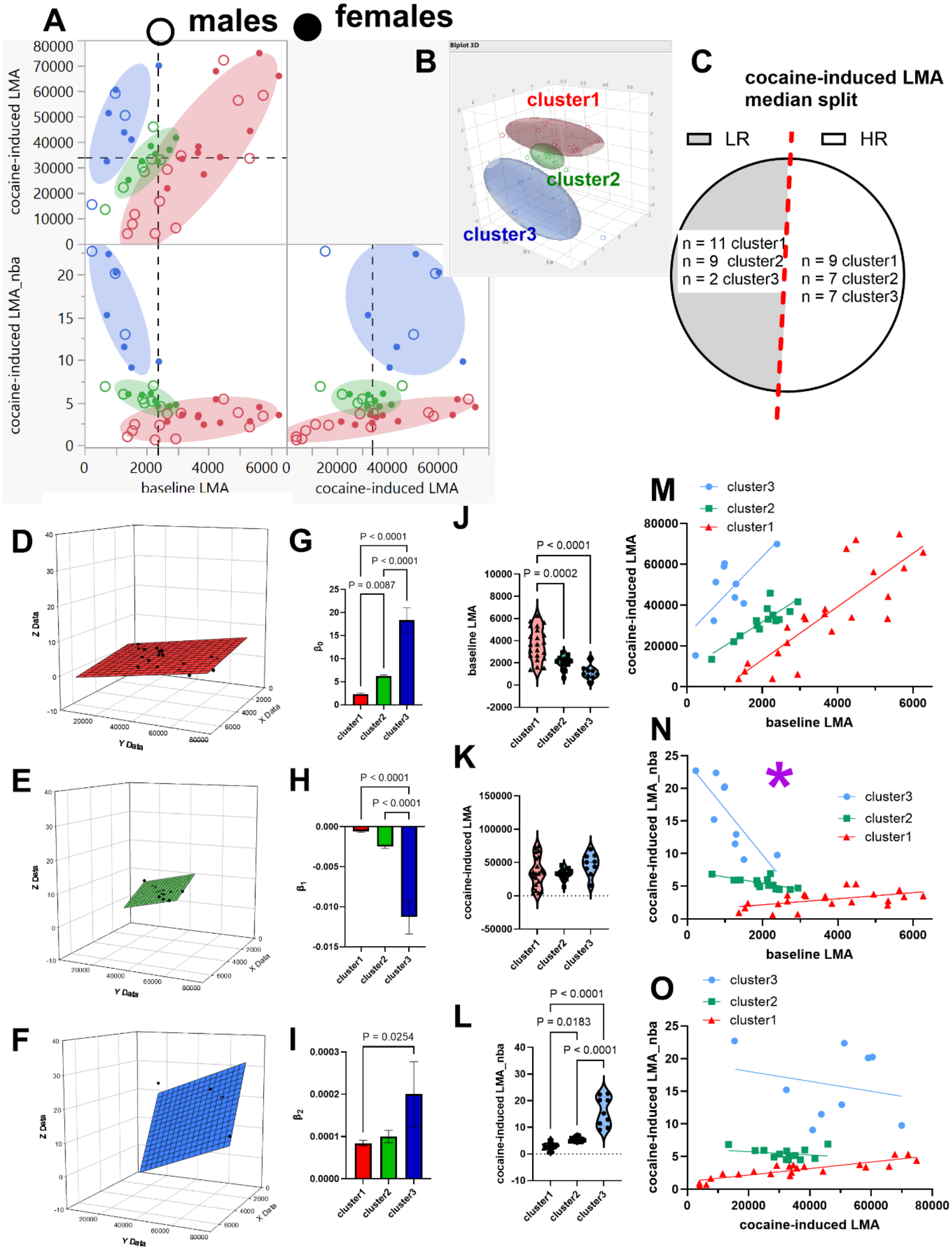
A
males
females
cocaine-induced LMA
cocaine-induced LMA_nba
baseline LMA
cocaine-induced LMA
B
cluster1
cluster2
cluster3
C
cocaine-induced LMA median split
LR
HR
n = 11 cluster1
n = 9 cluster2
n = 2 cluster3
n = 9 cluster1
n = 7 cluster2
n = 7 cluster3
D
E
F
G
H
I
J
K
L
M
N
O
cluster1
cluster2
cluster3
P < 0.0001
P = 0.0087
P = 0.0002
P = 0.0183
P = 0.0254
baseline LMA
cocaine-induced LMA
cocaine-induced LMA_nba

Figure S6

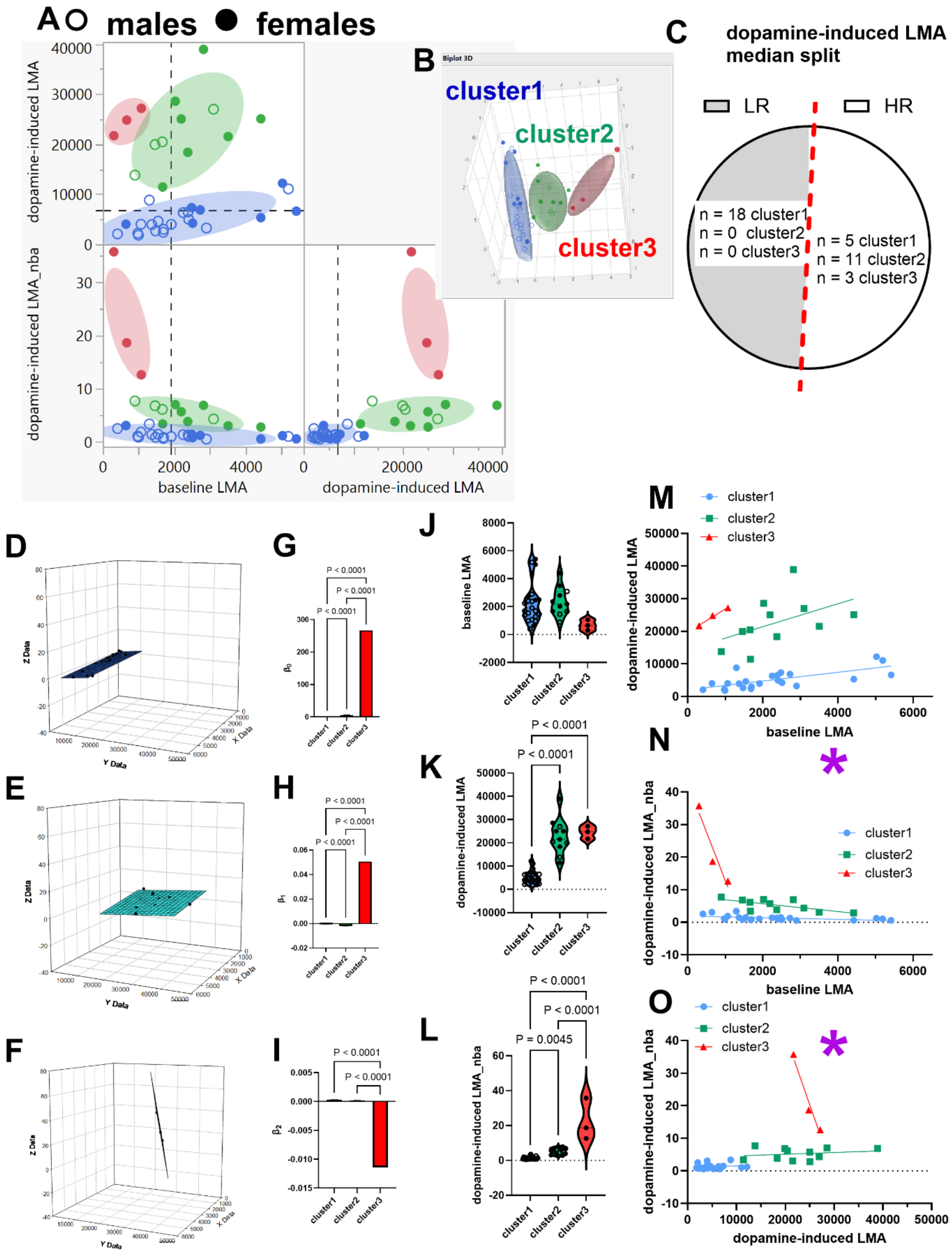

A males females
dopamine-induced LMA
dopamine-induced LMA_nba
baseline LMA
dopamine-induced LMA
B
cluster1
cluster2
cluster3
C dopamine-induced LMA median split
LR HR
n = 18 cluster1
n = 0 cluster2
n = 0 cluster3
n = 5 cluster1
n = 11 cluster2
n = 3 cluster3
D
E
F
G
H
I
J
K
L
M
N
O
P < 0.0001
P = 0.0045
baseline LMA
dopamine-induced LMA
dopamine-induced LMA_nba
cluster1
cluster2
cluster3

Figure S7

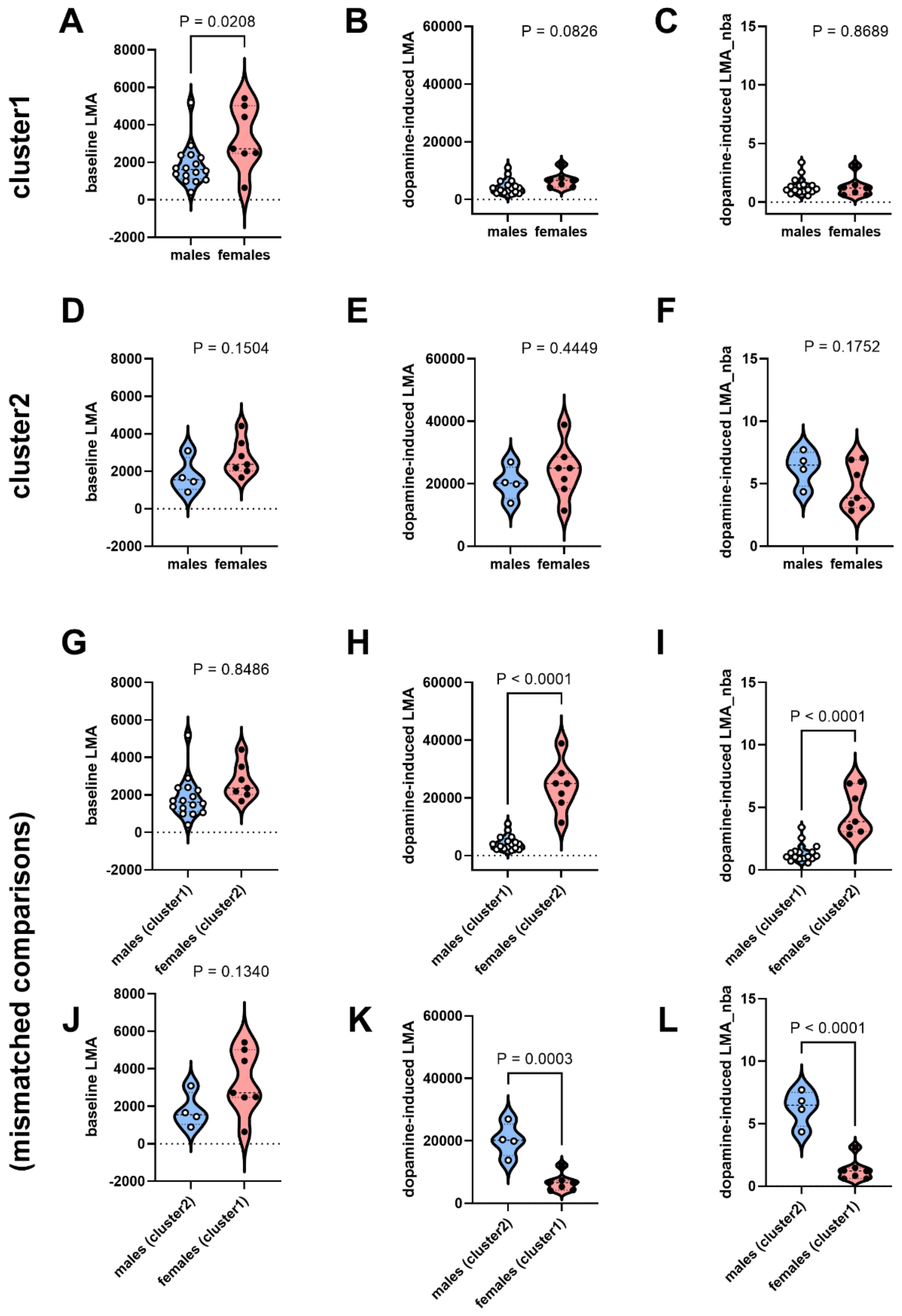
A
B
C
cluster1
P = 0.0208
P = 0.0826
P = 0.8689
baseline LMA
dopamine-induced LMA
dopamine-induced LMA_nba
males
females
D
E
F
cluster2
P = 0.1504
P = 0.4449
P = 0.1752
G
H
I
P = 0.8486
P < 0.0001
P < 0.0001
males (cluster1)
females (cluster2)
(mismatched comparisons)
J
K
L
P = 0.1340
P = 0.0003
P < 0.0001
males (cluster2)
females (cluster1)

Figure S8

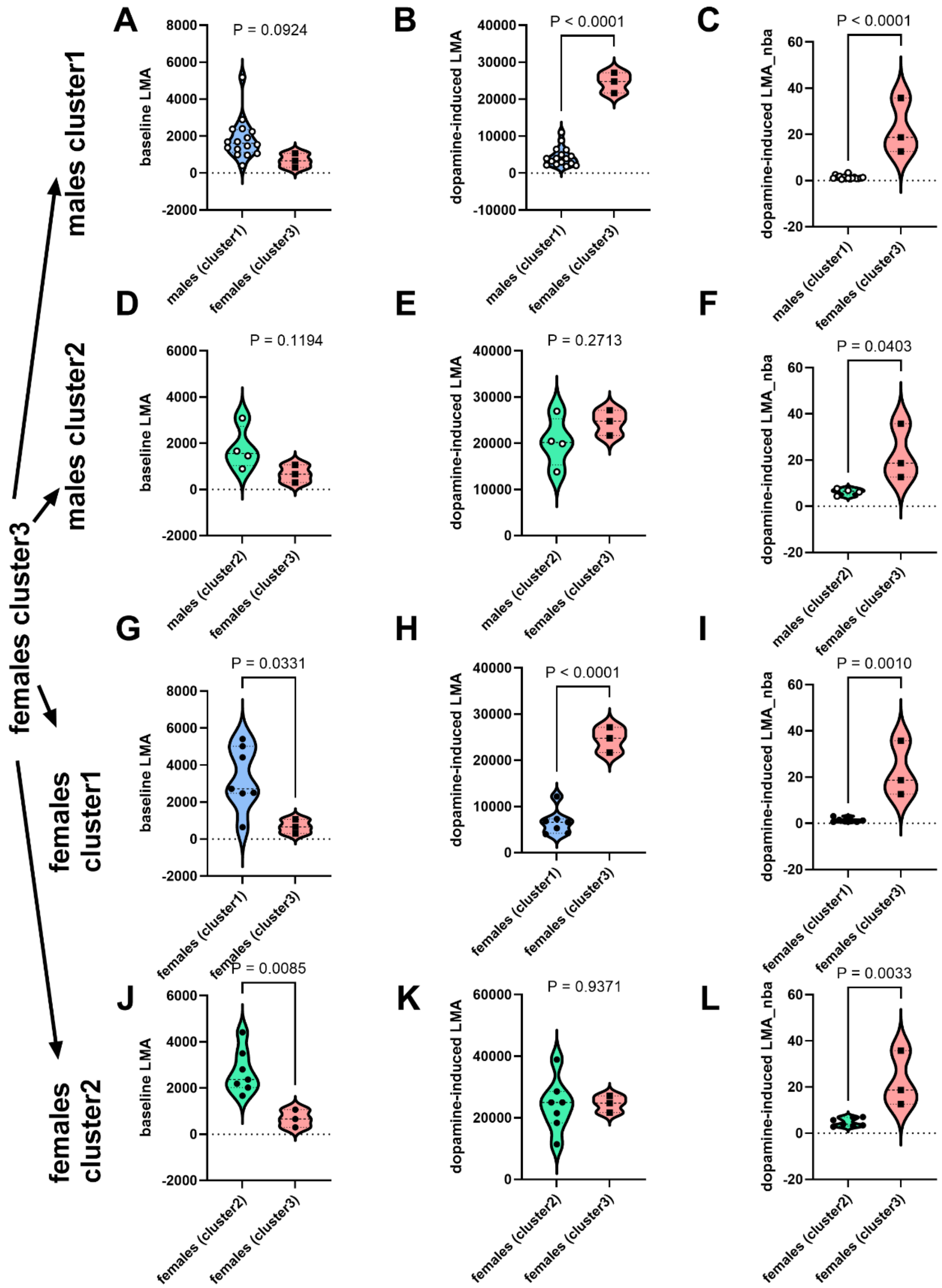

A
B
C
D
E
F
G
H
I
J
K
L
males cluster1
males cluster2
females cluster3
females cluster1
females cluster2
P = 0.0924
P < 0.0001
P < 0.0001
P = 0.1194
P = 0.2713
P = 0.0403
P = 0.0331
P < 0.0001
P = 0.0010
P = 0.0085
P = 0.9371
P = 0.0033
baseline LMA
dopamine-induced LMA
dopamine-induced LMA_nba
males (cluster1)
males (cluster2)
females (cluster1)
females (cluster2)
females (cluster3)

Figure S9
